# The association between accelerometer-measured physical activity and symptoms of depression and anxiety in children and adolescents: a systematic review and meta-analysis

**DOI:** 10.1186/s12887-025-06420-y

**Published:** 2025-12-28

**Authors:** Lin Wang, Alan R. Barker, Brad Metcalf, Yihao Liu, Melvyn Hillsdon, Lisa Price

**Affiliations:** 1 Faculty of Health and Life Sciences, Public Health and Sport Sciences, University of Exeter, Room 33 Haighton Library, Heavitree Road, St Luke’s Campus, Exeter, Devon EX1 2LU UK; 2 Faculty of Health and Life Sciences, Children’s Health and Exercise Research Centre, Public Health and Sport Sciences, University of Exeter, Exeter, UK; 3 Department of Social Psychiatry, West China Hospital, Sichuan University, Sichuan 610041 Chengdu, China

**Keywords:** Activity behaviours, Mental health, Young people, Accelerometery methodologies

## Abstract

**Background:**

Physical activity (PA) is associated with symptoms of depression and anxiety in children and adolescents. Using accelerometers to measure PA can provide more accurate and detailed data to investigate this relationship. However, no study has systematically reviewed and pooled the effects of accelerometer-measured PA on mental health. Therefore, this meta-analysis examined the association between accelerometer-measured PA and symptoms of depression and anxiety in children and adolescents and determined whether the strength of this relationship varied by accelerometery methodology.

**Methods:**

A systematic review conducted up to May 2025 using the following databases: Medline, PsycINFO, Embase, Web of Science, and SPORTDiscus, following the PRISMA guidelines, and using terms relating to children, PA, depression/anxiety and observational design. Meta-analyses were performed separately for 12 studies with continuous outcomes (Partial *r*) and 7 studies with binary outcomes (Odds Ratio). Subgroup analysis tested the moderating effects of accelerometer data collection and processing methods (e.g., epoch length, wear location, valid days, valid hours).

**Results:**

Thirty studies met the inclusion criteria (*n* = 15 cross-sectional, *n* = 15 prospective) including 40,334 youth aged 5–18 years. There was a small negative association of moderate-to-vigorous physical activity (MVPA) with depression (partial *r* = -0.17, 95%CI [-0.28, -0.06], *p* < 0.001) and anxiety (partial *r* = -0.21, 95%CI [-0.34, -0.09], *p* < 0.001). Total physical activity (TPA) (OR = 0.98, 95%CI [0.96,1.00], *p* = 0.05) and light intensity PA (OR = 0.95, 95%CI [0.92,0.98],* p* < 0.001) were associated with reduced risk of depression. The association between MVPA and depression appeared to vary by epoch length (≤ 15 s partial *r* = -0.32, 95% [-0.49, -0.15]; > 15 s partial *r* = -0.08, 95% [-0.19, 0.03]; difference *p* = 0.02). Sensitivity and publication bias analyses supported the overall robustness and reliability of the associations between PA and depression or anxiety.

**Conclusions:**

This review highlights that while PA was associated with reduced depression and anxiety in youth, substantial methodological heterogeneity, particularly in accelerometer protocols, may influence effect sizes. Improved and standardised accelerometry methodologies and developed innovative PA metrics are needed to better assess these relationships.

**Trial registration:**

The protocol for the present review was registered on PROSPERO (CRD42022320410) on 23rd March 2022.

**Key points:**

• This systematic review included 30 studies involving 40,334 children and adolescents aged 5–18 years and revealed substantial variability in accelerometer data collection and processing methods across studies, including 11 different device models, 4 wear locations, 6 epoch lengths, 4 intensity thresholds units, 5 valid day definitions, and 7 non-wear criteria, as well as 17 questionnaires used to assess depression and anxiety.

• Meta-analysis results showed that moderate-to-vigorous, total, and light physical activity were associated with reduced depression and anxiety in youth, while epoch length and accelerometer wear location may affect the magnitude of this relationship. The findings remained consistent across sensitivity analyses, and no evidence of publication bias was detected.

• Of the 30 studies assessed for risk of bias, 2 were rated as good quality, 22 as fair quality, and 6 as poor quality. The main quality problems were the lack of sample size justification, or insufficient reporting of uniform selection criteria.

**Supplementary Information:**

The online version contains supplementary material available at 10.1186/s12887-025-06420-y.

## Background

The prevalence of depression and anxiety in children and adolescents has seen a significant rise in recent years [1, 2]. In the UK, from 2017 to 2022, children and adolescents aged 6 to 17 years old reporting symptoms of mental disorders, including depression and anxiety, rose from 11.6% to 18% [3]. Furthermore, in 2023, 20.3% of 8 to 16 year olds reported having a probable mental health disorder [4]. According to global data, approximately 13% of adolescents aged 10 to 19 years old reported symptoms of mental disorders, predominantly depression and anxiety [5]. Symptoms of depression and anxiety in children and adolescents increase the risk of cardiovascular disease[6] and are negatively associated with brain health [7], well-being, quality of life [8], academic performance [9], social communication [10], and self-esteem [11].

Physical activity (PA)[12] is recommended to promote health and well-being in children and adolescents [13–15], and this extends to the prevention and management of depression and anxiety [16, 17]. Commonly used self-report methods for measuring PA, may either over or underestimate the true relationship with health outcomes as a result of social desirability bias and recall bias [18–20]. High resolution accelerometer data provides more detailed characterisation of the frequency, duration, intensity, and volume of PA [21, 22, 23] and facilitates a more precise understanding of the association between PA and symptoms of depression and anxiety in children and adolescents [24].

Despite the reduced biases afforded through device measures, accelerometer data processing decisions can still result in the over or underestimation of PA. Factors such as epoch length [25], accelerometer thresholds for differentiating PA intensities, and definitions of valid wear time can significantly alter estimates of PA and as a result, influence the observed relationship with health outcomes [24, 26][27] (Migueles et al., 2019). Existing studies of PA and symptoms of depression and anxiety in children report a variety of accelerometer data processing decisions, including differing epoch lengths from 5 to 60 s [28–30]; valid wear time from 2 to 6 days [31–34], and different accelerometer cut-point thresholds for estimating PA intensity.

While previous reviews have documented the association between PA and have not exclusively [35–37], these have not exclusively focussed on accelerometer-derived measures of PA. Furthermore, it is currently unknown how accelerometer data processing decisions may alter the relationship between PA and depression and anxiety. Consequently, a comprehensive review of the relationship between accelerometer-measured PA and depression and anxiety in children and adolescents, particularly considering the impact of accelerometer methodologies on this relationship, could provide further evidence for PA guidelines aimed at preventing and reducing the incidence of depression and anxiety in these age groups.

The present study will use a systematic review and meta-analysis of cross-sectional and prospective studies to investigate: 1) the association between accelerometer estimates of PA with symptoms of depression and anxiety in children and adolescents; 2) whether the observed relationship is modified by accelerometer data processing methods.

## Methods

The protocol for the present review was registered on PROSPERO (CRD42022320410) on 23rd March 2022. This systematic review was reported following the PRISMA guidelines [38]. The PRISMA checklist is presented in Supplementary Material A.

### Search strategy

The search was conducted up to May 2025 using the following databases: Medline, PsycINFO, Embase, Web of Science, and SPORTDiscus. Search terms were divided into 4 groups (population, exposure, outcome, and study design) and were adjusted according to the respective databases’ Thesaurus and Medical Subject Headings (MeSH) terms [39] through Ovid. The terms are related to population(s) group: “children and adolescents” (e.g. youth, child*, young people. Adolescent*, boy, girl, juvenile, teen*, school-age), exposure(s) group: “PA with accelerometer” (e.g., exercise, sport, movement, activity*, fitness, motor activity*, physical effort, physical exertion, habitual activity, free-living and accelerometer), and outcome(s) group: “depression and anxiety” (e.g., depression, low mood, anhedonia, anxiety, stress, mental health, well-being, wellbeing). The full search strategy is provided in Supplementary Material B.

### Screening of articles and eligibility criteria

Study inclusion criteria were: 1) PA was measured using accelerometers; 2) studies were cross-sectional or prospective; 3) participants were aged 5–18 years, including baseline and follow-up stages in prospective studies; 4) depression and anxiety symptoms were assessed using self-report questionnaires or interviews; 5) articles were published in English as full articles; and 6) publications from inception of the database to end of May 2025. Exclusion criteria were: 1) PA measured using subjective methods or objective methods other than accelerometers; 2) intervention studies (e.g., randomised controlled trials); 3) commentaries, conference abstracts, review articles, or theses; 4) participants aged over 18 years or below 5 years; and 5) depression and anxiety symptom assessments using mental health questionnaires without subscales for depression, anxiety, or emotional symptoms.

### Study selection

Data were formatted in RIS format and managed using Rayyan software [40]. After the searches, all duplicate articles were removed before reviewing titles and abstracts. Articles not fitting the inclusion criteria were then excluded, and full-text versions of articles that met the screening criteria were obtained. Two authors independently conducted the screening and selection process. For conflicting results or disagreements, a third reviewer was consulted to facilitate discussions and arrive at a consensus. The reference lists of included studies were thoroughly examined for forward and backward citation chasing to ensure the comprehensiveness and accuracy of the search. Inter-rater reliability for the title and abstract screening stage was assessed using Cohen’s kappa, based on inclusion and exclusion decisions made independently by two reviewers. The computed kappa was 0.39, indicating acceptable agreement [41]. The calculation process is detailed in the Supplementary Materials C.

### Data extraction

The following five dimensions of data were extracted by the first reviewer: fundamental characteristics (e.g., author, publication year, country, population, sample, age, sex, and weight status); measurement characteristics (e.g., accelerometer processing methods, depression, and anxiety assessment); covariates (e.g., age, sex, behaviour variables); statistical analyses (e.g., data analysis method), and effect size (e.g., including the measure of association between PA and depression/anxiety symptoms, 95% confidence intervals, and *p*-values). A second reviewer conducted an inspection of the data that had been preliminarily extracted on a sub-sample. If the required information was not presented in the article or supplementary files, an email was sent to the corresponding authors to request the missing information. If there was no response, a reminder email was sent, and if no reply was received, only the information provided in the paper was presented.

### Sources of heterogeneity and potential effect modifiers

We considered accelerometer-related methodological factors as potential effect modifiers, given their potential to influence PA estimates and associations with health outcomes. Many validation and calibration studies have demonstrated that differences in epoch length, wear location, and valid days can affect the estimation of PA intensity and total volume [42–44], which may have contributed to the heterogeneity across studies. For example, Rowlands et al. [45] investigated how epoch length (1-s vs. 60-s) in RT3 accelerometers affects the measurement of physical activity in 25 children aged 7–11 years. Each child wore two accelerometers simultaneously for six hours, and activity time across five intensity levels. Results showed that the 60-s epoch significantly overestimated moderate and vigorous activity, while it severely underestimated very hard activity, especially in highly active children. Yet, no previous review has examined whether such methodological variations impact the strength of associations with mental health. Thus, we included accelerometry characteristics in our moderator analysis to address this gap in the literature.

### Data synthesis

Data synthesis includes both narrative synthesis and meta-analysis. The narrative synthesis primarily provides a comprehensive description of each study's characteristics and methodologies, highlighting the extent and frequency of accelerometer methodology use. Meta-analysis guidelines were followed [46–48] to synthesise the relationship of PA with depression and anxiety symptoms in children and adolescents. Cross-sectional and prospective studies were synthesised and analysed into a single forest plot and stratified by study design. Additionally, the meta-analysis employed a random-effects model to accommodate the variability across studies [47, 49]. If the prospective studies reported a baseline relationship, this was also included in the analysis of the cross-sectional studies.

An assessment of the relationship between PA and depression and anxiety was undertaken using two different types of effect sizes (e.g., partial r, odds ratio). We chose to conduct separate meta-analyses for continuous and binary outcomes, to retain the original interpretability of the reported effect sizes and to minimize potential bias introduced by conversion procedures [47, 50]. For studies that analysed depression and anxiety on a continuous scale, the association statistics needed to be independent of (adjusted for) relevant covariates (age, sex, and BMI) based on partial correlation coefficients (partial *r* or standardised beta coefficients from a multiple linear regression [stdB], considered to be comparable [51].

Where authors only reported the ‘crude’ (unadjusted for covariates) association, a partial r was created as equation. Supplementary Material D show the partial *r* equation and convert effect).

Equation was employed to calculate an adjusted association based on estimates of the correlations between the covariates and both PA and depression/anxiety. For example, to ascertain the partial *r* between Moderate-to-vigorous physical activity (MVPA) and depression when controlling for age, ‘y x_1_’ denotes the estimated correlation between depression and MVPA, ‘y x_2_’ signifies the estimated correlation between depression and age, and ‘x_1_ x_2_’ denotes the estimated correlation between MVPA and age based on existing literature [33, 52, 53].

$${r}_{y{x}_{1}|{x}_{2}=\frac{{r}_{y{x}_{1}}-{r}_{y{x}_{2}}\cdot {r}_{{x}_{1}{x}_{2}}}{\sqrt{\left(1-{r}_{y{x}_{2}}^{2}\right)\left(1-{r}_{{x}_{1}{x}_{2}}^{2}\right)}}}$$

Equation 1: partial *r* calculation.

The direction of *r* was interpreted as being negative or positive, and the size was interpreted as a small, medium, and large effect using *r* ≥ 0.1, *r* ≥ 0.3, and *r* ≥ 0.5, respectively [54]. When studies reported an odds ratio (OR), data were combined using a meta-analytical approach that incorporates OR. An OR was considered significant if the 95% CI did not include 1, This meta-analysis used the adjusted odds ratio to combine incidence rates as the reported outcome [55]. Moreover, to effectively handle multiple indicators of the same constructs (e.g., different data points) within individual studies, we calculated an average effect size to represent the study in the meta-analysis [56].

Heterogeneity was assessed using the *I*^*2*^ statistic with percentages of 25% (*I*^*2*^ = 25), 50% (*I*^*2*^ = 50), and 75% (*I*^*2*^ = 75) used to indicate low, medium, and high heterogeneity, respectively [57]. To explore potential sources of heterogeneity, Aim 2 of the present study, which investigates the association between PA and symptoms of depression and anxiety in children and adolescents modified by choice of data processing methods, was undertaken through subgroup analysis. Subgroups were formed based on different accelerometer data processing methods, such as epoch length, wear location, and were stratified by study design (cross-sectional, prospective). Sensitivity analyses were performed to examine the robustness and reliability of the meta-analysis results, as well as to identify potential outlier studies [58]. This was accomplished using the 'leave-one-out' analysis method [59]. Possible publication bias was assessed using a funnel plot, if this plot was not symmetrical, then a new overall effect size, deemed to be adjusted for publication bias, is provided for comparison with the effect size of the published studies.

### Risk of bias

Two reviewers independently scored the risk of bias for all studies using the National Institutes of Health study quality assessment tool for observational cohort and cross-sectional studies [60]. Any discrepancies between the reviewers were resolved by consensus.

## Results

**Fig. 1:**
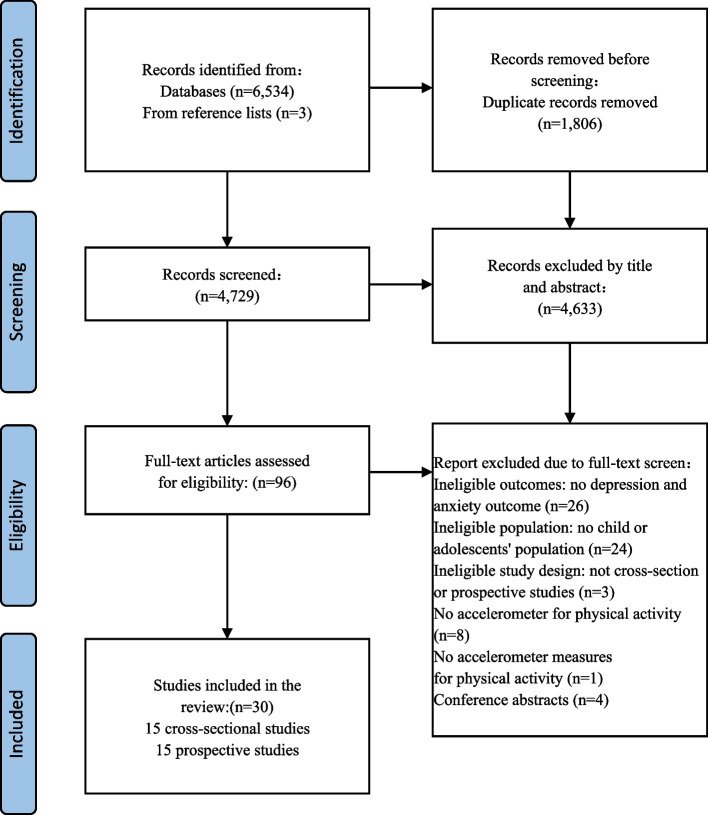
Flow chart of studies retrieved and screened following PRISMA guidelines

To ensure the inter-rater reliability of the study selection process, we assessed Cohen’s kappa during the initial screening. The resulting value (k = 0.39) indicates fair agreement, supporting the fairness and reliability of the subsequent article inclusion process. The search for this review was carried out up to April 2025. The outputs of these searches were combined, and an overview of the identification, screening, and eligibility processes is provided in Fig. 1. The overall search yielded a total of 6,534 articles, and an additional 3 articles were obtained through citation chasing. After removing 1,806 duplicates, a total of 4,728 articles remained. Following the screening of titles and abstracts, 4,633 articles were excluded. After reviewing the full texts, a total of 30 papers remained for review, consisting of 15 cross-sectional studies and 15 prospective studies.

### Participant characteristics

Table 1 shows the included cross-sectional studies (*n* = 15) with a total of 10,136 participants (range: 57 to 2,951), including 6,096 females (59.1%) and with a mean age of 13.2 (2.3) years old (range: 8 to 16 years). Studies were conducted in various countries: five from England, two from the USA, and one each from Iceland, Belgium, Iran, Australia, New Zealand, Sweden, Canada, and Brazil.

**Table 1:** Characteristics of the cross-sectional studies included in the review (*n* = 15)

	Study	Country	Sample size/female(n)	Mean age (SD)	BMI (SD) or BMI z score (SD)	Weight status (mean/SD or %)	Mean wear time per day/PA (min) Mean (SD)	Outcomes analysis and results	
								Depression/Emotion	Anxiety
1	Johnson et al. [61]	United States	1397/1397	12 (0.50)	20.77 (4.8)	NR	**MVPA**: 24 **VPA:** 6	MVPA (None) VPA (None)	
2	Parfitt, Pavey and Rowlands [62]	England	57/34	10.1 (0.3)	Male 16.5 (7.9) Female19.1 (8.9)	NR	**VLPA**: Male:224.4(43.0); Female: 231.2(120) **LPA**: Male:107.9(18.0); Female:120.0(15.6) **MPA:** Male:87.1(32.2); Female:87.6(17.1) **VPA:** Male:34.6(19.1); Female: 13.8(17.1)	VLPA (+) LPA(None) MPA (None) VPA (-)	VLPA (+) LPA(None) MPA(None) VPA (-)
3	Wiles et al. [34]	England	2951/1742	13.8 (0.2)	20.2 (3.3)	NR	**MVPA:** Overall: 21 **TPA:** Male: 319(median); Female: 281(median)	MVPA (-) TPA (-)	
4	Martikainen et al. [63]	England	199/107	8.1 (0.3)	NR	NR	**TPA(CPM):** Male:684.4(240.9); Female: 575.6(256.7) **MVPA:** Male: 28.2 (24.7); Female: 14.0(14.4)	TPA (-) MVPA (-)	TPA (-) MVPA (-)
5	Farren et al. [30]	United States	249/135	12.85 (0.89)	19.82 (3.18)	NR	**MPA:** Overall:166.08(50.91) **VPA:** Overall:22.79(16.47)	MPA (-) VPA (-)	
6	Hrafnkelsdottir et al. [64]	Iceland	244/144	15.8 (0.3)	21.9 (3.1)	Underweight: Total (244) = 19 (7.8), Male (100) = 8 (8.1), Female (144) = 11 (7.6), Normal: Total (244) = 193 (79.4), Male (100) = 83 (83.8), Female (144) = 110 (76.4), Overweight: Total (244) = 26 (10.7), Male (100) = 7 (7.1), Female (144) = 19 (13.2), Obese: Total (244) = 5 (2.1), Male (100) = 1 (1.0), Female (144) = 4 (2.8)	**TPA(CPM):** Overall:2039(472)	TPA(None)	TPA (None)
7	Hagemann et al. [65]	Belgium	934/588	13.27 (1.74)	NR	NR	**LPA:** Overall: 249.52(51.06), **MVPA:** Overall: 50.83(35.84)	LPA (-) MVPA (-)	LPA (-) MVPA (-)
8	Ghorbani et al. [33]	Iran	136/76	16.28(0.97)	23.28 (3.49)	NR	**MVPA:** Overall: 18.47(6.46) Boy: 19.66(7.10); Girl:16.96(5.22)	MVPA (-)	MVPA (-)
9	Dumuid et al. [66]	Australian	1182/579	12 (0.4)	NR	NR	**LPA:** Overall: 251(57) **MVPA:** Overall: 62(34)	NA	NA
10	Fairclough et al. [29]	England	359/180	11.5(1.4)	19.1 (3.6)	Underweight: (7.9%), Healthy: (67.6%), Overweight: (19.8%) Obese: (4.7%)	**LPA**: Overall: 210.1 **MVPA:** Overall: 50.9	LPA(None) MVPA(None)	
11	Costa., Chaput, et al. [67]	New Zealand	610/315	16.33 (1.04)	NR	Underweight: Total (610) = 10 (1.6), Female (315) = 6 (1.9), Male (295) = 4 (1.4) Healthy weight: Total = 451 (73.9), Female = 232 (73.7), Male = 219 (74.2) Overweight: Total = 106 (17.4), Female = 52 (16.5), Male = 54 (18.3) Obesity: Total = 43 (7.1), Female = 25 (7.9), Male = 18 (6.1)	**LPA:** Overall: 4.1 h Male: 4.03 h; Female: 4.38 h **MVPA:** Overall: 31.3(17.36) Male:33.30(19.39); Female: 29.42(14.99)	LPA (-) MVPA (-)	
12	Kjellenberg et al. [68]	Swedish	1139/580	13.4 (0.3)	0.36 (1.23)	Underweight: Total (1139) = 89 (7.8), Female (580) = 38 (6.6), Male (558) = 51 (9.2) Healthy weight: Total = 815 (71.8) Female = 430 (74.1), Male = 384 (69.3) Overweight: Total = 179 (15.8), Female = 89 (15.3), Male = 90 (16.2) Obesity: Total = 52 (4.6), Female = 23 (4.0), Male = 29 (5.2)	**MVPA** leisure time (weekdays): Overall: 31.6 (15.0); Male: 32.3(16.1); Female: 30.9 (13.8); **MVPA** leisure time (weekend): Overall: 37.9 (25.3); Male: 39.1(27.4); Female:36.8 (23.3) **MVPA** school time: Male:30.1(11.7); Female:26.5(11.2)		MVPA (-)
13	de Faria et al. [32]	Brazil	217/107	16 (NR)	21.2	NR	**LPA**: Overall:152.4(10.5) **MVPA**: Overall:52.8(3.7)	LPA (-) MVPA (None)	LPA (-) MVPA (None)
14	Costa., Bruner, et al. [69]	Canada	161/90	10.2 (2.30)	NR	NR	**LPA**: Overall: 209 (43.0) **MVPA:** Overall: 72.6 (30.6)	LPA (None) MVPA (None)	
15	Fairclough et al. [70]	England	301/181	11.1 (1.6)	19.1 (3.7)	Total 41.8 (12.8) Male = 41.5 (12.1) Female = 42.1 (13.3)	**LPA**: Male: 283.6 (250.4, 316.7); Female: 286.0 (253.2, 318.8) **MPA:** Male: 60.6 (54.7, 66.5); Female:48.9 (43.3, 54.6) **VPA:** Male:10.5 (9.1, 12.0); Female: 6.5 (5.1, 7.9)		

The signals in the outcomes analysed and main results columns indicate: (+) means positive relationship, (-) shows negative relationship, None presents no reported significant relationship between PA and depression or anxiety

*NR* Not report, *NA* Not available, *BMI* Body Mass Index, *LPA* Light Physical Activity, *MVPA* Moderate to Vigorous Physical Activity, *TPA* Total Physical Activity, *VPA* Vigorous Physical Activity

Table 2 shows prospective studies (*n* = 15) with 30,198 participants over 3.8 years (range: 1 to 8 years). Baseline ages were 5 to 14, with follow-up at 10 to 18 years. Gender was detailed in eight studies: 11,061 males and 7,299 females. Participants came from the UK, the Netherlands, Norway, Australia, New Zealand, Sweden, Finland, and the United States.

**Table 2:** Characteristics of the prospective studies included in the review (*n* = 15)

	Study	Country	Sample size/Female (%)	Sample size/female (%)	Mean age (range)	BMI (SD) or BMI z score (SD) baseline/follow up	Weight status (mean/SD or %)	PA Mean (SD)(Age)	Outcomes analysis and results
			(Baseline)	(Follow-up)					Depression/Emotion/Anxiety
1	Haapala et al. [71]	Finland	504	187/90	6–17	Baseline: 6–9 Follow up: 14–17	Overweight or obese 24% (12.8)	**MVPA:** 39 (20–68) min	LPA (None) MVPA (None)
2	Van Dijk et al. [72]	Dutch	158/97	158/97	13–16	Baseline: 18.75 (2.82) Follow up: 19.45 (2.83)	NR	**TPA:** Age:13.60(1.13)**:**699.09(187.86) Age: 14.62(1.20): 592.29(150.21)	Prospective: TPA (-)
3	Zahl et al. [73]	Norway	795 (NR)	8 years:699 (NR) 10 years:702 (NR)	6–10	Baseline: 15.60 (1.51) Follow up:16.62 (1.97)	NR	**MVPA:** 6 years: 1.19(0.40) hours 8 years:1.18(0.43) hours 10 years:1.09(0.40) hours	Prospective: MVPA (-) (6 to 8 years) MVPA (-) (8–10 years)
4	Ahn et al. [31]	England	6153/3159	6153/3159	7–11	NR	Not overweight: Male (2291) = 75.7%, Female (2289) = 70.2%, Overweight/obese: Male (650) = 24.3, Female (804) = 29.8	**LPA:** 7 years: Male: 282.83(37.67) Female: 279.46(39.69) **MVPA** 7 years: Male: 70.06(22.76) Female: 56.25(19.86)	Prospective: LPA(None) MVPA(None)
5	Bell et al. [28]	England	794/404	673/343	12—16	NR	NR	**TPA:** 12 years: Overall: 508.28(169.42) Boy: 552.31(174.35) Female: 465.93(153.25) **MVPA:** 12 years: Overall: 55.59(21.47) Boy: 61.56(22.07); Female:49.85(19.24)	Prospective: TPA (-) MVPA (-)
6	Hamer et al. [74]	England	4763/2524	2416/1280	7—14	Baseline: 16.5 (1.96) Follow up: NR	NR	**MVPA:** Low: 41(9) min/day; Medium: 60(9) min/day; High: 86(18) min/day **LPA**: Low: 260(38) min/day; Medium: 284(37) min/day; High: 299(37) min/day	Prospective: LPA of Girl (-) LPA of Boy (None) MVPA of boy and girl (None)
7	Kandola. et al. [16]	England	4257/2390	12 years: 2486/NR 14 years: 1938/NR 16 years: 1220/NR 18 years: 1128/NR	12—18	NR	Overweight or obese: 270/3671 (7.35)	**TPA:** 12 years:602.33 (177.62) cpm 14 years: 539.12 (181.43) cpm 16 years: 474.83 (158.68) cpm **LPA:** 12 year: 5.43 (0.97) hours 14 years: 4.64 (0.95) hours 16 years: 4.08 (0.92) hours **MVPA:** 12 years:23.279 (15.37) 14 years: 24.273 (16.91) 16 years: 23.523 (17.58) min	Anxiety: Prospective: LPA of 12 years (-) LPA of 14 years (-) LPA of 16 year (None) MVPA of 12 years (None) MVPA of 14 years (None) MVPA of 16 years (None)
8	Kandola. et al. [75]	England	4257/2390	12 years: 2486/NR 14 years: 1938/NR 16 years: 1220/NR 18 years: 1128/NR	12—18	NR	Overweight or obese (≥ 21 kg/m^2^): Total = 270/3671 (7·4%), normal weight or underweight (< 21 kg/m^2^): Total = 3401/3671 (92·6%)	**TPA: 1**2 years Overall: 603.33(177.62) Male: 662.04(184.92); Female: 547.97(151.51) 14 years: Overall: 539.12(181.43) Male: 598.61(192.38); Female: 486.67(153.15) 16 years: Overall: 474.83(158.68) Male: 529.90 (166.35); Female: 430.60(137.16) **LPA:** 12 years: Overall: 325.66(58.09) Male: 334.28(59.09); Female: 317.82(56.02) 14 years: Overall: 278.63(56.72) Male: 293.04(59.10); Female: 265.93(51.30) 16 years: Overall: 236.31(50.76) Male: 255.67(58.32); Female: 236.31(50.76) **MVPA:**12 years Overall:20 (11.86) Male:25.67(16.29); Female:15.59(9.67) 14 years: Overall: 20.71(12) Male: 25.82(15.34); Female: 16.8(9.67) 16 years: Overall: 19.5(10.25) Male: 25.69(16.8); Female: 15(7.17)	Cross-sectional: TPA of 12 years (-) LPA of 12 years (-) MVPA or 12 years (None) Prospective: TPA of 12 years (-) TPA of 14 years (-) TPA of 16 years (None) LPA of 12 years (-) LPA of 14 years (-) LPA of 16 years (-) MVPA of 12 years (-) MVPA of 14 years (None) MVPA of 16 years (None)
9	Slykerman, Budd, et al. [76, 77]	New zealand	871/442	467/248	11—16	Baseline: 19.1 (3.6)	NR	**MPA(NR)** **VPA(NR)**	Cross-sectional: MVPA(None), VPA(None) Prospective: MVPA(None), VPA(None)
10	Booth et al. [78]	England	4755/2627	3098/2137	11—13	BMI Z score 11 years: Male 0.34 (1.17) Female 0.27 (1.17) 13 years: Male 0.27 (1.18) Female 0.18 (1.16)	Baseline: Healthy weight Male = 1512 (71.4%), Female = 1917 (73.5%) Overweight Male = 288 (13.6), Female = 399 (13.0) Obese Male = 140(12.8), Female = 139(10.0)	**MVPA:** 11 years: Male: 29(17); Female: 18(12) 13 years: Male:29(19); Female:20(15)	Cross-sectional: MVPA (-) Prospective: MVPA(None)
11	Hume. et al. [79]	Australian	155/93	155/93	14—16	NR	Not overweight: Male = 73.8 Female = 78.5 Overweight: Male = 23.0 Female = 18.3 Obese: Male = 3.3 Female = 3.2	**MVPA:** Male:55.9(21.80); Female: 39.2(19.04) **VPA:** Male: 7.2(8.01); Female:3.5(4.16)	Cross-sectional: MVPA(None) VPA(None) Prospective: MVPA(None) VPA(None)
12	Nyberg et al. [80]	Sweden	1139/NR	585/321	13–14	NR	Underweight and normal weight = 467 (80.2) overweight and obesity = 115 (19.8)	**LPA:** 140.3(29.1) **MVPA:** 52.3(19.1)	LPA(None) MVPA(None)
13	Kracht et al. [17]	United States	330/180	205/111	10–16	Baseline: 23.9 (7.8) Follow-up: 26.0 (8.6)	Baseline: Underweight 3.4% Normal 13.2% Overweight 32.7% Obese 66.8% follow-up: underweight = 2.4% Normal = 14.2% Overweight = 33.7% Obese = 44.9%	**MVPA:** 29.8 (20.6)	MVPA (-)
14	Yang, Corpeleijn and Hartman. [81]	Netherlands	1070/NR	1070/NR	5–11	Baseline: 5.11 (0.89) Follow-up: 10.57 (0.55)		LPA: 264.71 (38.20) MPA: 43.75 (34.81, 54.80) VPA: 16.56 (11.23,24.19)	
15	Monteagudo et al. [82]	Spain	197/91	197/91	13–16	Baseline: 13.9. (0.3) Follow-up: 15.85 (0.3)		LPA: Female 171.42 (25.02) Male 176.14 (66.37) MVPA: Female 80.44 (23.96) Male 96.65 (30.14)	

The signals in the outcomes analysed and main results columns indicate: (+) means positive relationship, (-) shows negative relationship, None presents no reported significant relationship between PA and depression or anxiety

*NR* Not report, *NA* Not available, *BMI* Body Mass Index, *LPA* Light Physical Activity, *MVPA* Moderate to Vigorous Physical Activity, *TPA* Total Physical Activity, *VPA* Vigorous Physical Activity

### Data processing of accelerometer characteristics

Accelerometry methodology characteristics, including device type, wear location, epoch length, and cut-point, were available from 15 cross-sectional studies and 15 prospective studies, as detailed in Tables 3 and 4, respectively. The frequency of usage for each accelerometry methodology is provided in Table 5.

**Table 3:** Accelerometer data collection and processing methods for cross-sectional studies (*n* = 15)

Study	Device used *n* = 15	Wear location *n* = 14	Epoch size (seconds) *n* = 12	Cut point (counts, METs, or mg per minute) *n* = 27	Valid days (days) *n* = 14	Valid duration per day (hours) *n* = 10	Measurement period (hours) *n* = 10	Measurement period (days) *n* = 14	Weekend included in valid day (Yes/No) *n* = 14	Reason for non-wear *n* = 7	Non-wear validated *n* = 8	Mean wear time per day *n* = 7
1 Johnson et al. [61]	ActiGraph MTI	Hip	30	Sedentary: < 50 per 30 s LPA: 51–1499 per 30 s MPA: 1500–2600 per 30 s VPA: > 2600 per 30 s	6	NR	Waking time	6	No	Sleep and water-based	NR	NR
2 Parfitt, Pavey and Rowlands [62]	ActiGraph RT3	Hip	60	VLPA:100.0–470.1 LPA: 470.1–976.8 MPA 976.8–2337.2 VPA: > 2337.2	4	10	NR	7	Yes	NR	NR	NR
3 Wiles et al. [34]	Actiwatch AW4	Hip	60	LPA: 200–3,599, MPA:3,600–6,199 VPA: > 6,200	3	10	Awake time	7	No	NR	Zero counts ≥ 10 min	790.6 (55.8)
4 Martikainen et al. [63]	Actiwatch AW4	Wrist	60	MVPA > 2297	4	NR	NR	7	Yes	NR	NR	NR
5 Farren et al. [30]	Actical accelerometers	Wrist	60	Sedentary: < 0.01 kcal/kg/min LPA: 0.01 ≤ AEE < 0.04 kcal/kg/ MVPA: 0.04 ≤ AEE < 0.10 kcal/kg/	4	13	Waking time	5	No	Sleep and Water-based	NR	896.82 (81.69)
6 Hrafnkelsdottir et al. [64]	ActiGraph GT3X	Wrist	60	Group median value	4	14	24	7	Yes	NR	NR	NR
7 Hagemann et al. [65]	Fitbit Charge 2	Hip	60	MVPA: ≥ 3 METs	3	8	24	7	No	Water-based	Zero value for all PA intensity	NR
8 Ghorbani et al. [33]	ActiGraph GT3X	Hip	NR	NR	3	8	Waking time	7	No	Sleep and water-based	Zero counts ≥ 90 min	NR
9 Dumuid et al. [66]	GENEActiv	Wrist	60	Sedentary: 0–6 g per 1 s LPA: 6–21 g per 1 s MPA: 22–25 g per 1 s VPA: > 56 g per 1 s	4	10	24	7	No	NR	NR	1432 (30)
10 Fairclough et al. [29]	ActiGraph GT9X	Wrist	5	LPA: 50 mg per 1 s MVPA: 200 mg per 1 s	3	16	24	7	No	NR	Value range at least 2 out of the 3 axis being less than 13 and 50 mg or value range was less than 50 mg	22.8(1.0) hour per day
11 Costa., Chaput, et al. [67]	ActiGraph GT3X and wGT3x +	Wrist	5	Sedentary: 35.6 mg per 1 s LPA: 35.6–201.4 mg per 1 s MVPA ≥ 201.4 mg per 1 s	4	16	24	7	Yes	Water-based	NR	NR
12 Kjellenberg et al. [68]	Actigraph GT3X	Hip	5	Sedentary: 0–100 MVPA ≥ 2296	3	8	Waking time	7	Yes	Water-based	Zero counts ≥ 60 and no spike tolerance was used	792.6 (60.8)
13 de Faria et al. [32]	Actigraph GT3X	Hip	15	NR 2	6	10	24	8	Yes	Water-based	Zero counts ≥ 20 min	1376
14 Costa., Bruner, et al. [69]	GENEActiv	Wrist	5	NR	4	16	More than 16 but less than 24	8	NR	NR	Value range at least 2 out of the 3 axis being less than 13 and 50 mg or value range was less than 50 mg	13.5 (1.3) hour per day
15 Fairclough et al. [70]	ActiGraph GT9X	Wrist	5	LPA: 48 mg per second MPA: 201 mg per second VPA: 707 mg per second	3	16	24	7	Yes	NR	Value range at least 2 out of the 3 axis being less than 13 and 50 mg or value range was less than 50 mg	22.8(1.0) hour per day

The signals in the outcomes analysed and main results columns indicate: (+) means positive relationship, (-) shows negative relationship, None presents no reported significant relationship between PA and depression or anxiety

*NR* Not report, *NA* Not available, *BMI* Body Mass Index, *LPA* Light Physical Activity, *MVPA* Moderate to Vigorous Physical Activity, *TPA* Total Physical Activity, *VPA* Vigorous Physical Activity

**Table 4:** Accelerometer data collection and processing methods for prospective studies (*n* = 15)

Study	Device used *n* = 11	Wear location *n* = 10	Epoch size (seconds) *n* = 9	Cut point (counts, METs, or mg per minute) *n* = 22	Valid days (days) *n* = 10	Valid duration per day (hours) *n* = 8	Measurement period (hours) *n* = 9	Measurement period (days) *n* = 10	Weekend included in valid days (Yes/No) *n* = 10	Non-wear of device *n* = 7	Non-wear validated *n* = 4	Mean wear time per day *n* = 0
1 Haapala et al. [71]	Actiheart (CamNtech Ltd)	Chest	60	MET-based: < 1.5 (SB), 1.5–4 (light), 4–7 (moderate), > 7 (vigorous)	4	12	24	4	Yes	NR	NR	NR
2 Van Dijk et al. [72]	ActivPAL3	Thigh	NR	NR	4	NR	24	6	Yes	NR	NR	NR
3 Zahl et al. [73]	ActiGraph GT3X	Wrist	10	Sedentary: ≤ 100 MVPA ≥ 2296	3	8	24	7	No	Water-based	Zero counts ≥ 20 min	NR
4 Ahn et al. [31]	ActiGraph GT1M	Wrist	15	Sedentary < 100 LPA: 100–2240 MVPA: 2241–11,714,	2	10	Waking time	7	No	Water-based	Zero counts ≥ 20 min	NR
5 Bell et al. [28]	ActiGraph GT1M	NR	10	NR (Referenced by Evenson (2008))	3	8	16	7	No	Water-based	Zero counts ≥ 60 min	NR
6 Hamer et al. [74]	ActiGraph GT1M	Wrist	5	Sedentary: < 100 LPA: 100–2241 MVPA: > 2241	2	10	12	7	No	Water-based	NR	NR
7 Kandola. et al. [16]	Actigraph MTI	Hip	60	Sedentary ≤ 199 LPA: 200–3599 MVPA: ≥ 3600	3	10	Waking time	7	No	Water-based	NR	NR
8 Kandola. et al. [75]	Actigraph MTI	Hip	60	Sedentary: ≤ 199 LPA: 200–3599 MVPA: ≥ 3600	3	10	Waking time	7	No	Water-based	NR	NR
9 Slykerman, Budd, et al. [76, 77]	Actigraph AM	Hip	NR	Sedentary: < 3 METs (0–1135 count) MPA: 3 to 6 METs (1136–3908) VPA: ≥ 6 METs (3909–20000)	NR	NR	Waking time	7	NR	NR	NR	NR
10 Booth et al. [78]	Actigraph AM	Hip	60	MVPA > 3600	3	10	Waking time	7	No	NR	Zero counts ≥ 10 min	NR
11 Hume. et al. [79]	Actigraph AM	Hip	60	LPA: ≤ 50 counts/min MPA ≥ 3 METs VPA ≥ 6 METs,	4	NR	Waking time	7	No	NR	NR	
12 Nyberg et al. [80]	Actigraph, GT3X +	Hip	5	Sedentary 0- 100 LPA 101–2295 MVPA ≥ 2296 (Evenson et al., 2011)	3	8	Waking time	7	Yes	Water-based activities	Zero counts ≥ 60 min	NR
13 Kracht et al.[17]	Actigraph, GT3X +	Hip	15	Sedentary 0- 100 LPA 101–2295 MVPA ≥ 2296 (Evenson et al., 2011)	4	10	NR	7	Yes	Water-based activities	NR	NR
14 Yang, Corpeleijn and Hartman. [81]	ActiGraph GT3X	Hip	15	Sedentary ≤ 819 cpm LPA 820–3907 cpm MPA 3908–6111 cpm VPA ≥ 6112 cpm	3	10	Waking time	NR	Yes	Water-based activities	Zero counts ≥ 90 min	NR
15 Monteagudo et al. [82]	GENEActiv	Non-dominant wrist	1	Sedentary: 0–6 g per 1 s LPA: 7–19 g per 1 s MVPA: ≥ 20 g per 1 s	4	24	24	NR	Yes	Water-based activities	NR	NR
15												

The signals in the outcomes analysed and main results columns indicate: (+) means positive relationship, (-) shows negative relationship, None presents no reported significant relationship between PA and depression or anxiety

*NR* Not report, *NA* Not available, *BMI* Body Mass Index, *LPA* Light Physical Activity, *MVPA* Moderate to Vigorous Physical Activity, *TPA* Total Physical Activity, *VPA* Vigorous Physical Activity

**Table 5:** The frequency for data processing characteristics of accelerometer cross-sectional and prospective studies (*n* = 30)

Data processing metrics of accelerometer	Frequency (n)	References	Data processing metrics of accelerometer	Frequency (n)	References
**Cross-sectional studies**			**Prospective studies**		
Device used			Device used		
*ActiGraph MTI*	1	[61]	*ActiGraph MTI*	2	[75, 83]
*RT3*	1	[62]	Actiheart	1	
*Actiwatch AW4*	2	[34, 63]			
*Actical accelerometers*	1	[30]			
*Fitbit Charge 2*	1	[65]			
*ActiGraph GT3X*	5	[32, 33, 64, 67, 68]	*ActiGraph GT3X*	4	[17, 73, 80, 81]
*GENEActiv*	2	[69, 84]	*GENEActiv*	1	[82]
*ActiGraph GT9X*	2	[29, 70]			
			*ActiGraph GT1M*	3	[28, 31, 74]
			*Actigraph AM*	3	[76–79]
			*ActivPAL3*	1	[72]
Wear location			Wear location		
*Hip*	7	[32–34, 61, 62, 68]	*Hip*	8	[16, 17, 75–81]
*Wrist*	8	[29, 30, 63, 64, 66, 67, 69, 70]	*Wrist*	4	[28, 31, 73, 82]
			*Thigh*	1	[72]
			*Chest*	1	[71]
Epoch size (seconds)			Epoch size (seconds)		
			1	1	[82]
*5*	5	[29, 67–70]	*5*	2	[74, 80]
			*10*	2	[28, 73]
*15*	1	[32]	*15*	3	[17, 31, 81]
*30*	1	[61]			
*60*	7	[30, 34, 62–64, 66]	*60*	4	[78, 79,]^79^,^83^,^75^
Not report	*n* = 1	[33, 65]	Not report	2	[72, 76, 77]
Cut point (reference) (CPM, METs, mg, or ACC) *n* = 44			Cut point (reference) (CPM, METs, mg, or ACC)		
*CPM*	*n* = 10		CPM	n = 20	
*LPA*	3 thresholds		*LPA*	4 thresholds	
	51–1499 per 30 s (*n* = 1)	[61]			
	470.1–976.8 per min (*n* = 1)	[62]			
	200–3599 per min (*n* = 1)	[34]		200–3599 per min (*n* = 2)	[75, 83]
				100–2241 per min (*n* = 2)	[31, 74]
	≤ 50 per min (*n* = 1)			100–2295 per min (*n* = 2)	[17, 80]
				820–3907 per min (*n* = 1)	[81]
*MPA*	2 thresholds		*MPA*	1 thresholds	
	1500–2600 per 30 s (*n* = 1)	[61]		3908–6111 per min (*n* = 1)	[81]
	976.8–2337.2 per min (*n* = 1)	[62]			
	3600–6199 per min (*n* = 0)				
*MVPA*	1 thresholds		*MVPA*	4 thresholds	
	≥ 2297 per min (*n* = 1)	[63]		2241–11,714 per min (*n* = 1)	[31]
				> 2241 per min (*n* = 1)	[74]
				≥ 3600 per min (*n* = 3)	[75, 78, 83]
				≥ 2296 per min (*n* = 3)	[17, 73, 80]
*VPA*	3 thresholds		*VPA*	1 thresholds	
	> 2600 per 30 s (*n* = 1)	[61]			
	> 2337.2 per min e(n = 1)	[62]		≥ 6112 per min (*n* = 1)	[81]
	> 6200 (*n* = 1)	[34]			
*SED*	1 thresholds		*SED*	2 thresholds	
	< 50 per 30 s (*n* = 1)	[61]			
				≤ 100 per min (*n* = 4)	[17, 31, 74, 80]
				≤ 199 per min (*n* = 2)	[75, 83]
mg	*n* = 9				
*LPA*	3 thresholds		*LPA*	1 thresholds	
	6–21 g per second (*n* = 1)	[84]		7–19 g per second (*n* = 1)	[82]
	50 mg per second (*n* = 1)	[29]			
	LPA: 48 mg per second (*n* = 1)	[70]			
	35.6–201.4 mg per min (*n* = 1)	[67]			
*MPA*	1 threshold		*MPA*		
	22–25 per second (*n* = 1)	[84]			
	201 mg per second (*n* = 1)				
*MVPA*	2 thresholds		*MVPA*	1 thresholds	
	200 mg per min (*n* = 1)	[29]		≥ 20 g per second (*n* = 1)	[82]
	> 201.4 mg per min (*n* = 1)	[67]			
*VPA*	1 threshold		*VPA*		
	56 mg per second (*n* = 1)	[84]			[82]
	LPA: 48 mg per second (*n* = 1)	[70]			
*SED*	2 thresholds		*SED*	1 thresholds	
	6 g per second (*n* = 1)	[84]		> 7 g per second (*n* = 1)	[70]
	35.6 mg per second (*n* = 1)	[67]			
*METs*	*n* = 1		*METs*	*n* = 3	
			*SED*	< 1.5	[71]
			*LPA*	1.5–4	[71]
*MPA*	thresholds		*MPA*	thresholds	
				3–6 per min (*n* = 1)	[76, 77]
				4–7 per min (*n* = 1)	[71]
				≥ 3 per min (*n* = 1)	[79]
*MVPA*	1 threshold		*MVPA*		
	≥ 3 per min (*n* = 1)	[65]			
*VPA*			*VPA*	1 thresholds	
				≥ 6 per min (*n* = 2)	[76, 77, 79]
				> 7 per min (*n* = 1)	[71]
*ACC*	*n* = 3		*ACC*		
*LPA*	1 threshold		*LPA*		
	0.01 ≤ AEE < 0.04 kcal/kg/per min (*n* = 1)	[30]			
*MVPA*	1 threshold		*MVPA*		
	0.04 ≤ AEE < 0.10 kcal/kg/per min (*n* = 1)	[30]			
*SED*	1 threshold		*SED*		
	< 0.01 kcal/kg/min per min (*n* = 1)	[30]			
Not report	*n* = 4	[32, 33, 64, 69]	Not report		[28, 72]
Valid days			Valid days		
			*2*	2	[31, 74]
*3*	6	[29, 33, 34, 64, 68, 70]	*3*	7	[28, 73, 75, 78, 80, 81, 83]
*4*	7	[30, 62, 63, 65, 66, 69, 85]	*4*	4	[17, 71, 72, 79, 82]
*6*	2	[32, 61]			
			Not report	1	[76, 77]
Valid duration per day (hours)			Valid duration per day (hours)		
*8*	2	[33, 68]	*8*	3	[28, 73, 80]
*10*	4	[32, 34, 62, 66]	*10*	6	[17, 31, 74, 75, 78, 83]
*13*	1	[30]			
*16*	4	[29, 67, 69, 70]			
Not report	4	[61, 63–65]	Not report	4	[72, 76, 77, 79]
Measurement period (hours)			Measurement period (hours)		
			*12*	1	[74, 75, 78, 83]
			*16*	1	[28]
*24*	7	[29, 32, 64–67, 70]	*24*	4	[71–73, 82]
Waking time	5	[30, 33, 34, 61, 68]	Waking time	7	
More than 16 but less than 24	1	[69]			
Not report	2	[62, 63]	Not report	1	[17]
Measurement period (days)			Measurement period (days)		
*5*	1	[30]			
*6*	1	[61]	*6*	1	[72]
*7*	11	[29, 33, 34, 62–68, 70]	*7*	11	[17, 28, 31, 73–75, 78–80, 83]
*8*	2	[32, 69]	Not report	1	[81]
Weekend included in valid days			Weekend included in valid days		
*Yes*	7	[32, 62–64, 67, 68, 70]	*Yes*	5	[17, 71, 72, 80, 81]
*No*	7	[29, 30, 33, 34, 61, 65, 66]	*No*	8	[28, 31, 73–75, 78, 79, 83]
Not report	1	[69]	Not report	1	[76, 77]
Non-wear of device			Non-wear of device		
*Water-based*	*4*	[32, 65, 67, 68]	*Water-based*	9	[16, 17, 28, 31, 73–75, 80–82]
*Sleep and water-based*	*3*	[30, 33, 61]			
Not report	8	[29, 34, 62–64, 66, 69, 70]	NR	3	[72, 76, 77, 79]
Non-wear validated			Non-wear validated		
*Zero counts* ≥ *10 min*	1	[34]	*Zero counts* ≥ *10 min*	1	[78]
*Zero counts* ≥ *20 min*	1	[32]	*Zero counts* ≥ *20 min*	2	[31, 73]
*Zero counts* ≥ *60 min*	1	[68]	*Zero counts* ≥ *60 min*	1	
*Zero counts* ≥ *60 min, with evctor magnitude and no spoke tolerance*			*Zero counts* ≥ *60 min, with evctor magnitude and no spoke tolerance*	1	[80]
*Zero counts* ≥ *90 min*	1	[33]	*Zero counts* ≥ *90 min*	1	[81]
*Zero value for all PA intensity*	1	[65]			
*Value range at least 2 out of the axis being less than 13 or or value range was less than50 mg*	3	[29, 69, 70]			
Not report	7	[30, 61–64, 66, 67]	Not report	7	[70, 72, 74–77, 79, 83]

*LPA* Light Physical Activity, *MVPA* Moderate to Vigorous Physical Activity, *TPA* Total Physical Activity, *VPA* Vigorous Physical Activity, *SED* Sedentary, *CPM* Counts per minute, mg:, *METs* Metabolic equivalents, *ACC* Activity energy expenditure

### Cross-sectional studies

The 15 cross-sectional studies used 7 different models of accelerometer, with ActiGraph GT3X being commonly used in 5 studies. The wrist was the preferred wear location in 8 studies. 12 studies reported epoch lengths, with the 60-s epoch most common (*n* = 7). Fourteen studies identified 10 cut-point thresholds, categorised by counts per minute (cpm), milli-g (mg), and metabolic equivalent and activity energy expenditure thresholds corresponding to energy expenditure using accelerometer counts. Cpm was used most frequently (*n* = 6), with intensities threshold references to Treuth, Adolph and Butte [86] and Evenson et al. [87], 3 studies used mg with the Hildebrand et al. [88] cut-points. One study applied ≥ 3 METs for moderate to vigorous physical activity (MVPA) with the Brewer, Swanson and Ortiz [89] cut-point, and another used < 0.01 kcal/kg/min for sedentary, 0.01 ≤ AEE < 0.04 for light physical activity (LPA), and 0.04 ≤ AEE < 0.10 for MVPA, following the Romanzini et al. [90] cut-point. Four studies used compositional data analysis [29, 32, 66, 70].

The measurement duration protocol varied across studies, with a wear duration of 5–8 days and an expected wear time of either 24 h or during waking hours. Inclusion criteria for analysis required between 3–6 valid days and 8–16 valid hours per day. Specifically, 11 studies followed a 7-day measurement protocol, with 6 studies using a 24-h and another 5 studies using a waking time protocol. A 4-day period was the most common criteria for valid days (*n* = 8 studies), while a 10-h daily minimum was most frequent criteria for valid hours (*n* = 4 studies). Additionally, 7 studies included at least 1 weekend day in their analysis. Eight studies defined non-wear duration using 7 different definitions, including ≥ 10, ≥ 20, and ≥ 60 consecutive zero counts without spike tolerance, while one study applied a zero value across all measured variables. Another study followed Choi et al. [91] ≥ 90 zero counts definition. Three studies used criteria for two axes: a threshold of less than 13 mg for one axis and less than 50 mg for the other [92]. Additionally, one study applied Ridley's method, imputing activity percentages for non-wear [93]. Five studies reported that data could include non-wear periods based on water-based actives, such as swimming or bathing. Mean daily wear time reported ranged from 790.6 to 1432.0 min across 7 studies.

### Prospective studies

The 15 prospective studies used 5 different models of accelerometer, with ActiGraph GT3X, GT1M, and AM being commonly used. The hip was the preferred location for accelerometer placement (*n* = 8). Epoch varied from 5 to 60 s, with 60 s epochs most common (*n* = 5). Eight studies defined PA intensity with counts, often using Pulsford et al. [94], Mattocks et al. [95], and Evenson et al. [87] cut-points, while 2 studies measured intensity in METs, primarily using Trost, Way and Okely [96] thresholds. 11 studies reported a 7-day measurement duration, with most using a waking hours protocol waking hours (*n* = 7). For valid days, most studies required 3 days of wear (*n* = 7), with 6 studies requiring at least 1 valid weekend day. Seven studies required 10 h of wear for a day to be classed as valid. Only 6 studies defined non-wear duration with 3 thresholds: ≥ 10 zero counts (*n* = 1), ≥ 20 zero counts (*n* = 2), ≥ 60 zero counts (*n* = 2), ≥ 90 zero counts (*n* = 1). Additionally, 9 studies reported that data could include non-wear periods based on water-based actives, such swimming or bathing.

### Outcomes measurement characteristics

In the reviewed studies, depression and anxiety were assessed using 15 different questionnaires and two interview assessments. As Table 6 shows, for anxiety, assessments included the State-Trait Anxiety Inventory (STAI-C; Spielberger, 1971), Clinical Interview Schedule-Revised (CIS-R anxiety scale; Lewis et al., 1992), and Spence Children's Anxiety Scale (SCAS-S; Spence, 1998). Depression assessments involved 10 distinct scales: the Centre for Epidemiological Studies–Depression scale (CES-D; Radloff, 1991), Child Depression Inventory (CDI; Smucker et al., 1986), Preschool Age Psychiatric Assessment (PAPA; Egger & Angold, 2004), Child and Adolescent Psychiatric Assessment (CAPA; Angold et al., 1995), the short version of the Mood and Feelings Questionnaire (SMFQ; OLD & RCPsych, 1995), Mood and Feelings Questionnaire (MFQ: Angold & Costello, 1987), Symptom Checklist-90 (SCL-90: Derogatis, Rickels, & Rock, 1976), Depression Anxiety Stress Scale-21 (DASS-21: Lovibond & Lovibond, 1995), Spanish version of the Behaviour Assessment System for Children and Adolescents (BSCA-SPANISH: González et al., 2004), Child Behaviour Checklist (CBCL: Achenbach, Edelbrock, & Howell, 1987), Beck Depression Inventory (BDI: Aaron, 1961) and the General Health Questionnaire (GHQ: DP, 1988). Also, the subscale of Strengths and Difficulties Questionnaire (SDQ: Goodman, 1997) was applied to examine combination of depression and anxiety symptoms.

**Table 6:** Summary of the depression and anxiety measurement methods

	Questionnaires or interview (outcomes)	Items/Point method	Range score/High risk (score)	Who reported	References
1	CES-D (depression)	16 items: four-point 20 items: four-point	0–48: > 24 0–60: ≥ 15 0–60: > 24 0–60: ≥ 20	Self-report NR Self-report Self-report	[30, 61, 67, 72, 76, 77, 79]
2	STAI-C (anxiety)	NR	0–16: high score	Self-report	[83]
3	MFQ (depression)	13 items: three-point 33 items: three-point	0–26: 0–2(low), 3–5(mid), > 6(high); 0–66: high score	Self-report	[29, 34]
4	CBCL (mix)	NR: two-point	NR: > 82%	Parents and teacher	[63]
5	CDI (depression)	27 items: NR	0–52: high score	Self-report	[62]
6	SCL-90 (mix)	10 items for depression 4 items for anxiety: five-point	NR: depression ≥ 30, anxiety ≥ 12	Self-report	[64]
7	BSI (mix)	8 items for depression, 5 items for anxiety: four-point	0–32 for depression, 0–25 for anxiety: high score	Self-repot	[65]
8	DASSL-21 (mix)	7 items for depression, 7 items for anxiety: three-point	0–21 for each: high score		[33]
9	SDQ (emotion)	5 items for emotion: 0–10	0–10: high score	Self-report Or parents (child below11 years)	[28, 31, 69, 70, 78, 81] [74](baseline)
10	SMFQ (depression)	13 items: three-point	0–16: > 12	Self-report	[75] (baseline)[78] [74] (follow-up)
11	SCAS-S (anxiety)	19 items: four-point	0–57: > 1SD(low risk), < 1SD(high risk)		[83]
12	GHQ (mix)	12 items: four-point	0–12: high score	Self-report	[32]
13	PAPA (depression)	NA	0–9 for depression	Interview	[73]
14	CAPA (depression)	NA	0–21 for anxiety	Interview	[73]
15	CIS-R (depression)	NR	0–21: NR	Self-report	[75] (follow-up)
16	BASC (depression & anxiety	41	0–180: < 60 for each (high risk)	Self-report	[82]
17	BDI (depression)	21 items: four-point	0–63	Self-report	[71]

MH Measurement questionnaires of depression and anxiety: Centre for Epidemiological Studies–Depression scale (CES-D), Centers for Epidemiological Studies Depression Scale for Children (CES-DC), Child Depression Inventory (CDI), Symptom Checklist-90 (SCL-90), Short version of the Mood and Feelings Questionnaire (SMFQ), Depression, Anxiety, Stress Scale-21 (DASSL-21), Brief Symptom Inventory-53, assessing general symptoms (BSI) State-Trait Anxiety Inventory anxiety symptoms (STAI-C), General Health Questionnaire (GHQ), Strengths and Difficulties Questionnaire (SDQ) Short version of the Spence Children’s Anxiety Scale (SCAS-S), Child Behaviour Checklist with the subscale of anxious/depressed (CBCL), Preschool Age Psychiatric Assessment (PAPA), Child and Adolescent Psychiatric Assessment (CAPA), CIS-R anxiety, Mood and Feelings Questionnaires (MFQ), Spanish version of the Behavior Assessment System for Children and Adolescents (BASC); Beck Depression Inventory (BDI)

### Risk of bias assessment

In the quality assessment of 15 prospective studies, one study was categorized as “Good [71]. 10 studies were rated as “Fair” [17, 28, 31, 73, 74, 78–82] and 4 studies [75–77, 79, 83] as “Poor”. The main quality issues were that no studies reported on the blinding of outcome assessors, the consistent implementation of outcome measures, and just half of the studies reported uniform selection criteria, repeated assessments of exposures over time and loss to follow-up in prospective studies. In cross-section studies, In assessing the quality of fourteen cross-sectional studies, one study was categorized as “Good” [32], 12 as "Fair” [29, 30, 33, 34, 61–65, 67, 68, 70] and 2 [69, 84] as "Poor" quality. The main quality issue was that only two studies [32, 65] reported sample size justification. Risk of bias data are provided in Supplementary Material E.

### Meta-analysis

#### Partial r

**Fig. 2:**
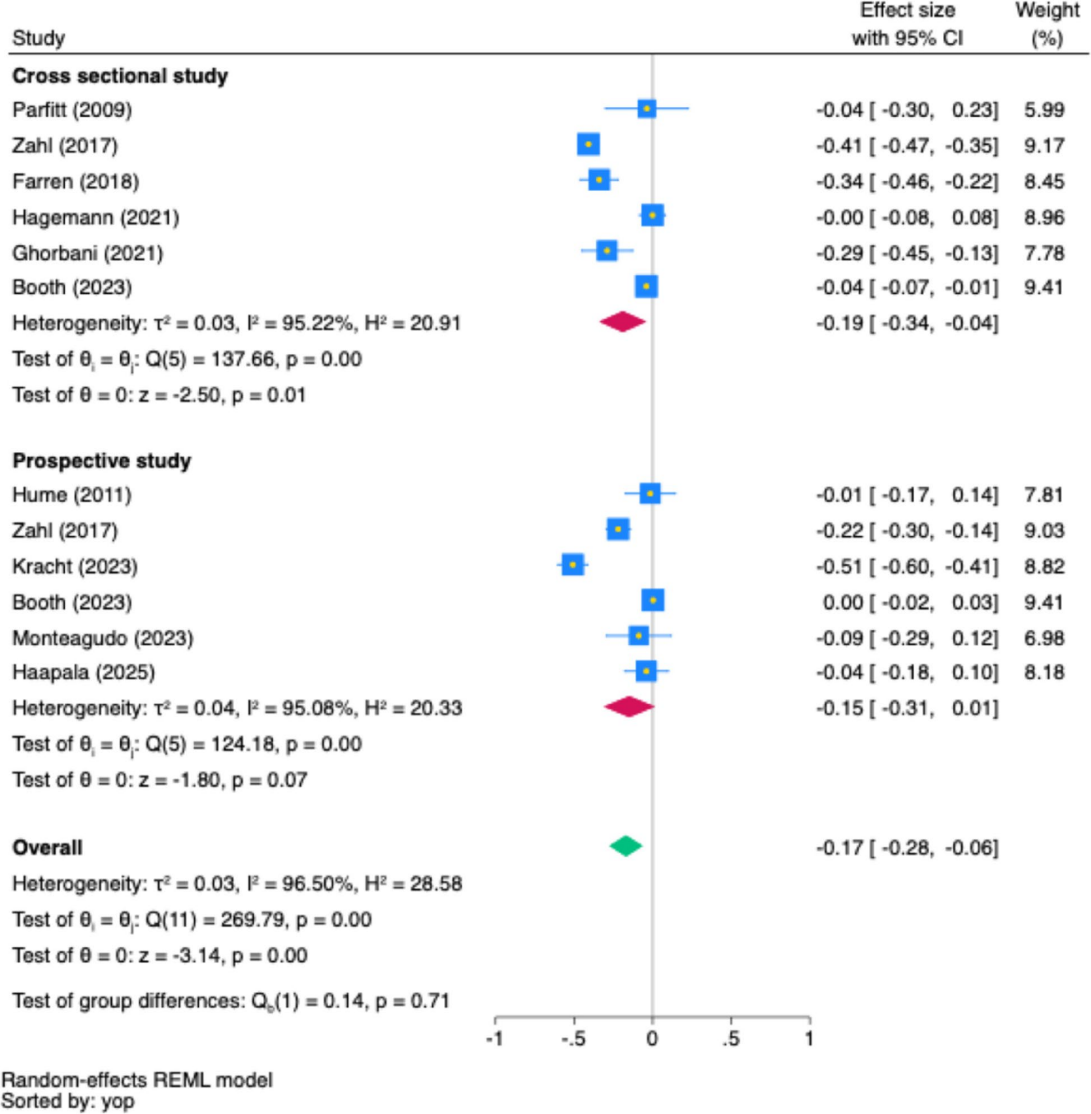
Pooled Association (Partial *r*) Between MVPA and Depression Across Study Designs. Note: In the forest plot, each square shows the effect size of a study, with its area reflecting the study's weight in the meta-analysis. The diamond represents the combined effect size estimate of all studies. The blue square represents the study's weight, its yellow midpoint indicates the effect size, while the blue width denotes the 95% confidence interval. Confidence intervals that cross the line of no association (partial *r* = 0.00) indicate a lack of statistical significance. yop: the sorted by the year of publication

Only MVPA had adequate effect size data for the data synthesis (Fig. 2) and was, therefore, the only intensity considered for partial *r* meta-analysis. In cross-sectional studies, MVPA had a small, negative association with depression (*r* = −0.19, 95%CI [−0.34, −0.04], *p* < 0.001, *n* = 7), with high heterogeneity (*I*^*2*^ = 95.22%, *p* < 0.01). No association between MVPA and depression in prospective studies (*r* = −0.15, 95%CI [−0.31, 0.01], *p* = 0.07, *n* = 6), with high heterogeneity (*I*^*2*^ = 95.10%, *p* < 0.001). When combining cross-sectional and prospective study designs, MVPA had a small, negative association with depression (*r* = −0.17, 95%CI [−0.28, −0.06], *p* < 0.001) with high heterogeneity (*I*^*2*^ = 96.5%, *p* < 0.001) and no design-based differences (*p* = 0.71). Due to the availability of sufficient effect size data, only MVPA related to anxiety that was considered in cross-section studies for data synthesis.

**Fig. 3:**
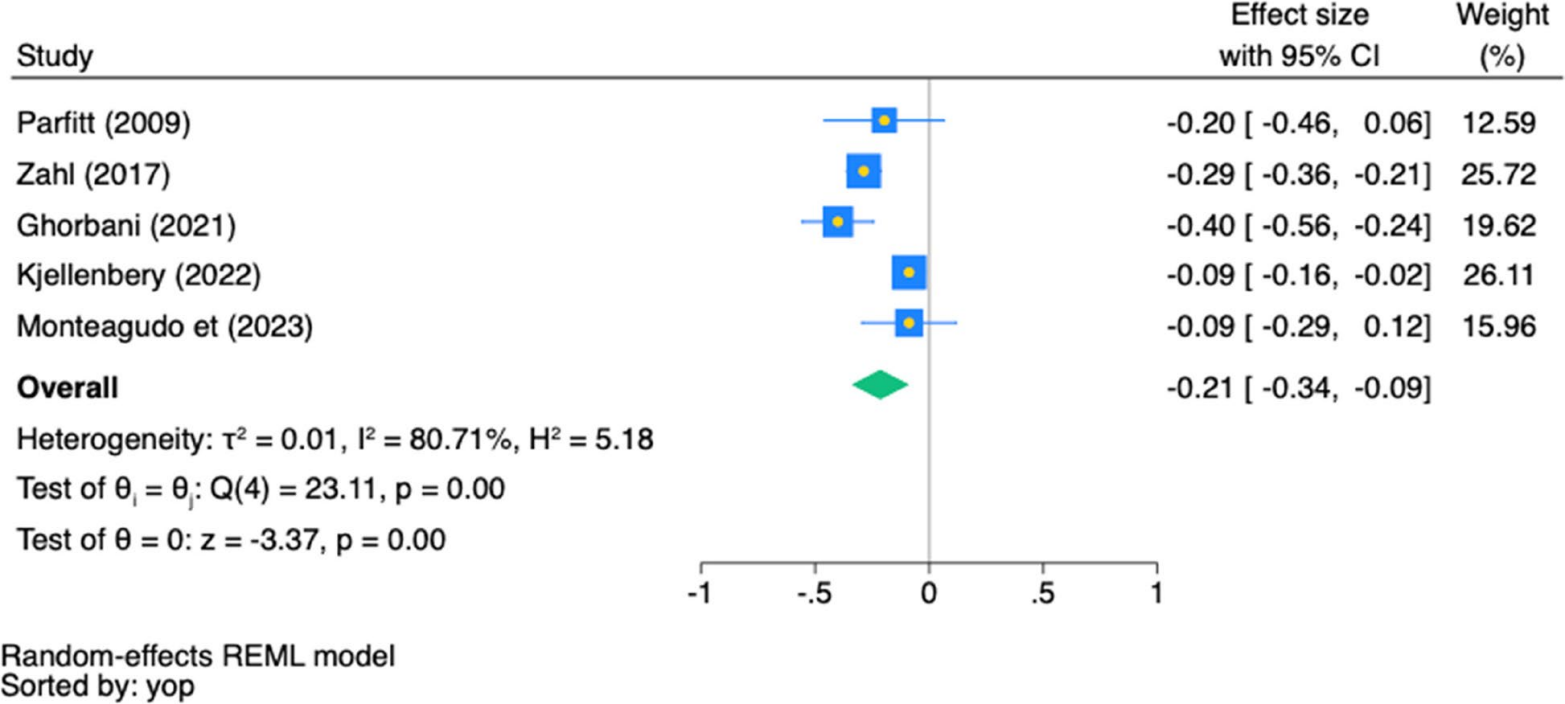
Pooled Association (Partial *r*) Between MVPA and Anxiety in Cross-sectional Studies. Note: In the forest plot, each square shows the effect size of a study, with its area reflecting the study's weight in the meta-analysis. The diamond represents the combined effect size estimate of all studies. The blue square represents the study's weight, its yellow midpoint indicates the effect size, while the blue width denotes the 95% confidence interval. Confidence intervals that cross the line of no association (partial* r* = 0.00) indicate a lack of statistical significance. yop: the sorted by the year of publication

Figure 3 illustrates that MVPA had a significant, negative association with anxiety (partial *r* = −0.21, 95% CI [−0.34, −0.09], *p* < 0.01), with high heterogeneity (*I*^*2*^ = 80.71%, *p* = 0.01) across the cross-sectional studies.

### Odds ratio

**Fig. 4:**
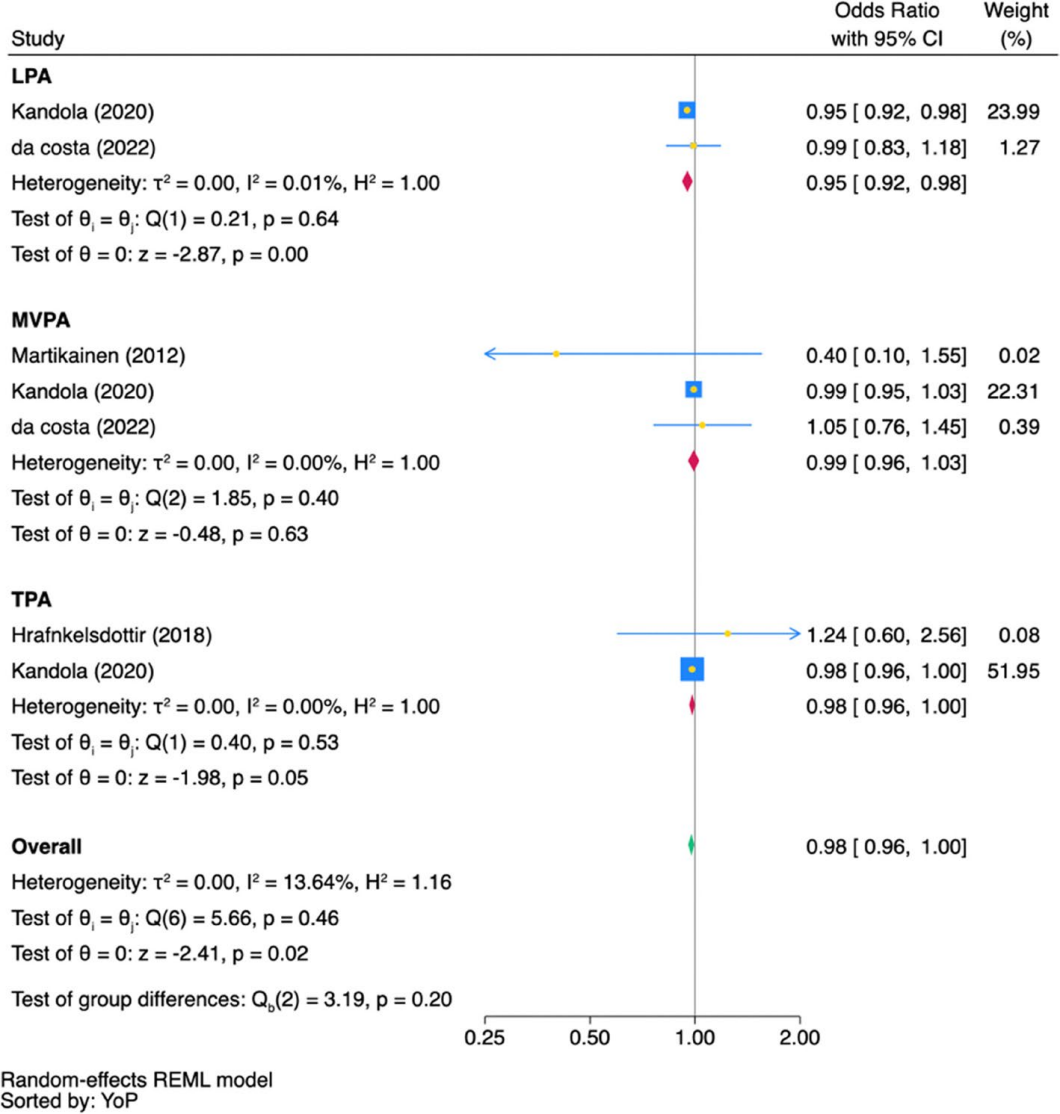
Fig. 4 Pooled Association (OR) Between PA Intensity and Depression in Cross-sectional Studies. Note: In the forest plot, each square shows the effect size of a study, with its area reflecting the study's weight in the meta-analysis. The diamond represents the combined effect size estimate of all studies. The blue square represents the study's weight, its yellow midpoint indicates the effect size, while the blue width denotes the 95% confidence interval. Confidence intervals that cross the line of no association (OR = 1.00) indicate a lack of statistical significance. YoP: the sorted by the year of publication

Figure 4 synthesises the OR from cross-sectional studies, assessing the likelihood of depression across different PA intensities. Light physical activity (LPA) and total physical activity (TPA) were significantly negatively associated with depression (LPA: OR = 0.95, 95% CI [0.92, 0.98]; *p* < 0.001, *n* = 2; TPA: OR = 0.98, 95% CI [0.96, 1.00],* p* = 0.05, *n* = 2).

### Exploring heterogeneity with subgroup comparisons

**Fig. 5:**
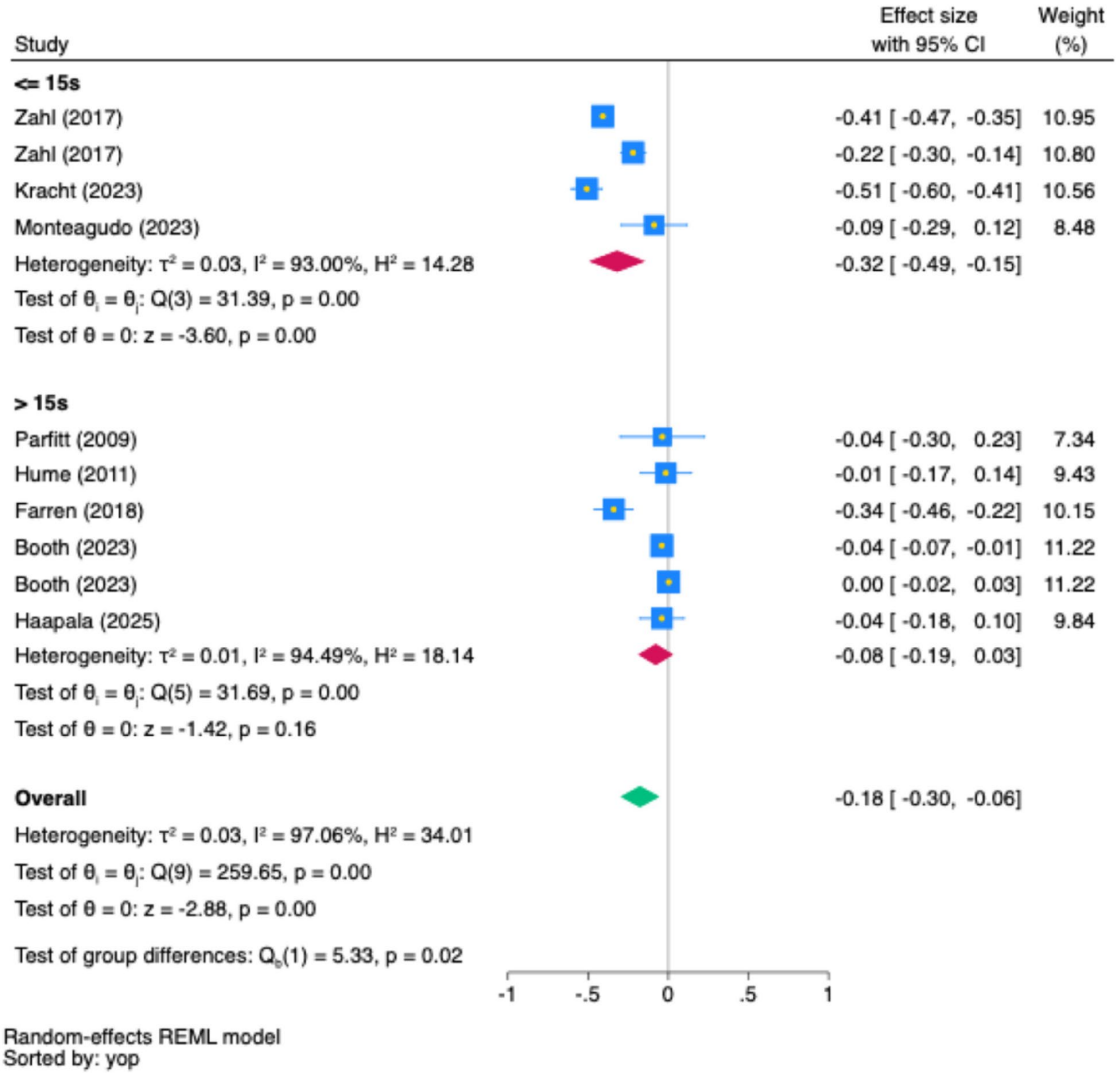
Subgroup Analysis of Epoch Length on MVPA and Depression Association (Partial *r*). Note: In the forest plot, each square shows the effect size of a study, with its area reflecting the study's weight in the meta-analysis. The diamond represents the combined effect size estimate of all studies. The blue square represents the study's weight, its yellow midpoint indicates the effect size, while the blue width denotes the 95% confidence interval. Confidence intervals that cross the line of no association (partial *r* = 0.00) indicate a lack of statistical significance. yop: the sorted by the year of publication

**Fig. 6:**
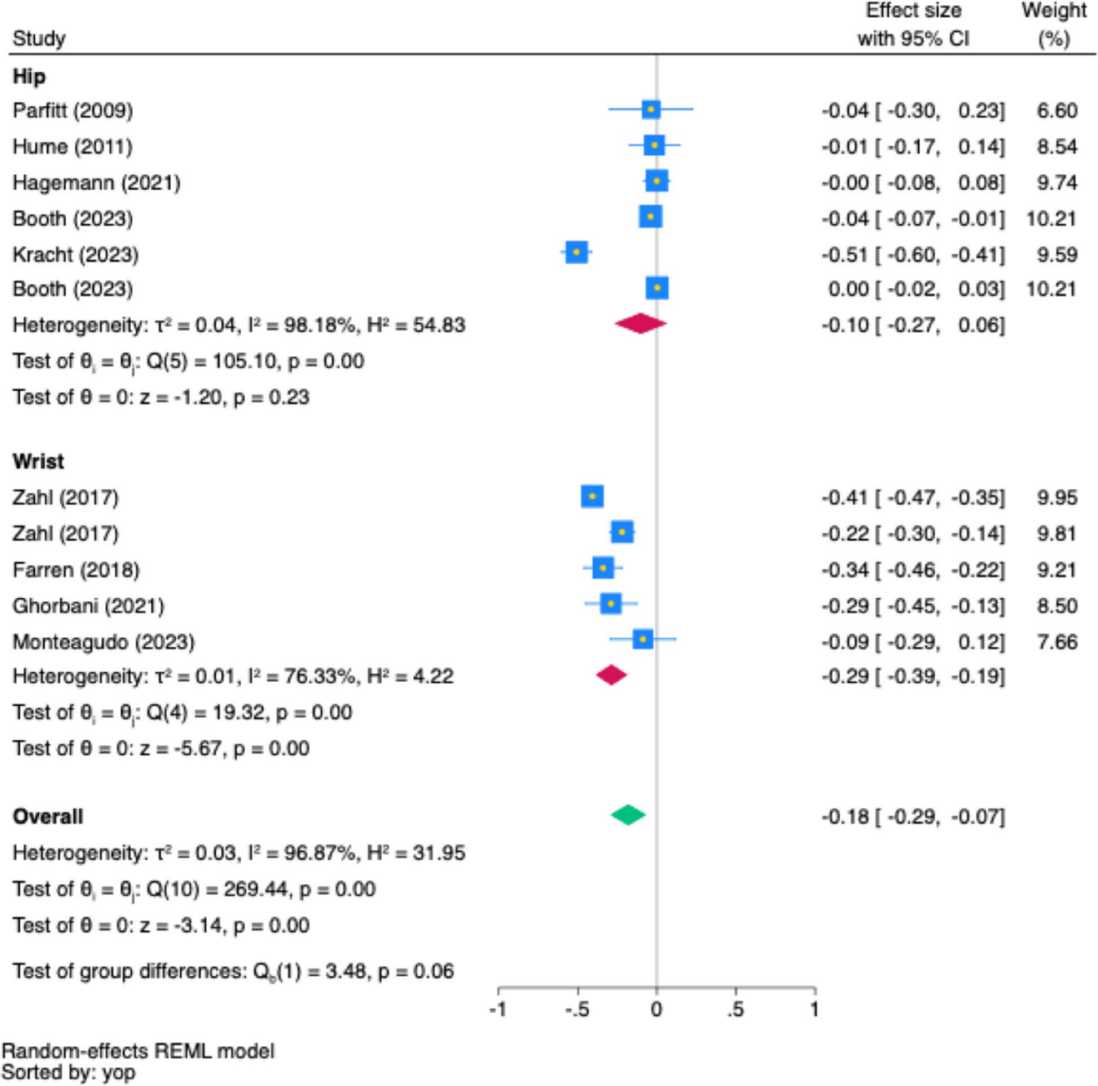
Subgroup Analysis of Wear Location on MVPA and Depression Association (Partial *r*)*.* Note: In the forest plot, each square shows the effect size of a study, with its area reflecting the study's weight in the meta-analysis. The diamond represents the combined effect size estimate of all studies. The blue square represents the study's weight, its yellow midpoint indicates the effect size, while the blue width denotes the 95% confidence interval. Confidence intervals that cross the line of no association (partial *r* = 0.00) indicate a lack of statistical significance. yop: the sorted by the year of publication

Subgroup analyses identified epoch length as a significant moderator and wear location as a marginally significant moderator, as illustrated in Figs. 5 and 6. Results of other subgroup analyses are presented in Supplementary Material F.

Figure 5 illustrates that the epoch length was identified as a significant moderating factor (Cochran’s Q = 5.33, *p* = 0.02). Studies using an epoch length ≤ 15 s presented a negative association between MVPA and depression (partial *r* = −0.32, 95% CI [−0.49, −0.15]; *I*^*2*^ = 93%, *p* < 0.001). In contrast, studies using an epoch length > 15 s showed no significant association between MVPA and depression (partial *r* = −0.08, 95% CI [−0.19, 0.03]; *I*^*2*^ = 94.49%, *p* < 0.001).

Figure 6 illustrates that the wear location was identified as a borderline significant moderating factor (Cochran’s Q = 3.48, *p* = 0.06). Studies using wrist-worn location showed a medium, negative association between MVPA and depression (partial *r* = −0.29, 95% CI [−0.39, −0.19]; *I*^*2*^ = 76.33%, *p* < 0.001). and small significant association between MVPA and depression was observed for hip-worn accelerometers (partial *r* = −0.10, 95% CI [−0.27, −0.06]; *I*^*2*^ = 98.18%, *p* < 0.001).

### Sensitivity analysis

Sensitivity analysis (Supplementary Material G) demonstrated a robust association between MVPA and depression or anxiety (partial *r*), with the negative relationship remaining statistically significant when employing the leave-one-out method. However, the association between PA and depression for the OR result showed slight inconsistency, with one study displaying a non-significant effect. Although the overall pattern still suggests a weak negative association. This minor variation indicates that while the results are largely reliable, they may be subject to influence by individual study effects.

### Publication bias analysis

The association between MVPA and depression (partial *r*) included 11 studies and required no imputations, suggesting an absence of publication bias and a robust, statistically significant negative effect size. In contrast, the association between MVPA and anxiety (partial *r*) initially showed a slight publication bias, with one study imputed to the right, but the negative effect size remained significant even after adjustment, underscoring the reliability of the findings. Lastly, the association between PA and depression for OR results also indicated a robust effect size, with the analysis not affected by publication bias, as the effect size remained consistent after including an imputed study. These findings affirm the stability and reliability of the reported associations in the presence of potential publication bias. The funnel plot shown as Supplementary Material H.

## Discussion

This systematic review with meta-analysis examined the relationship between accelerometer-measured PA and depression and anxiety symptoms in children and adolescents and further examined if this relationship was altered by accelerometry methodologies. The main findings of this study are: 1) The partial* r* effect showed that MVPA had a small negative association with symptoms of depression and anxiety; 2) For the OR effect, TPA and LPA had a small negative relationship with depression; 3) Epoch length significantly moderated the association between PA and depression, and wear location also showed a near-significant moderating effect.

Previous reviews that have investigated the association between PA and depression and anxiety symptoms in children and adolescents have shown similar results to those presented in this study [97, 98, 85, 35, 99, 100, 101, 102]. Ahn and Fedewa [97] conducted a meta-analysis including both intervention and observational studies (*n* = 19) involving participants aged 5 to 18 years. The study found a small but statistically significant negative association between PA and depression (*r* = −0.14, SE = 0.04). Korczak, Madigan and Colasanto [35] reported a small negative association between PA and depression symptoms (*r* = −0.14, [95% CI: −0.19 to −0.10]) when examining 28 cross-sectional studies and 12 prospective studies which used both objective and subjective PA measurement methods. Poitras et al. [37] reported a favourable relationship between device-measured TPA and depression in school-aged children based on the outcomes from 5 studies. Lu et al. [103]’s systematic review and meta-analysis synthesized 16 observational studies using sensor-based PA measurement and converted various reported effect sizes (e.g., relative risks) into odds ratios (ORs) for a unified analysis. An inverse association was found between TPA, MVPA, and Vigorous physical activity (VPA) and depressive symptoms, with odds ratios ranging from 0.73 to 0.83, but no such association was observed for LPA. Further, McDowell et al., [36] reviewed 2 studies that reported a negative relationship between self-reported PA and anxiety in children and adolescents, further supporting the findings of the current study. Despite all findings supporting a small to moderate negative association, our pooled effect sizes were slightly larger than those reported in previous meta-analyses (e.g., *r* = −0.17, [95% CI: −0.28, −0.06] for depression; *r* = −0.21, [95% CI: −0.34, −0.09] for anxiety). This may be because our synthesis focused specifically on MVPA, a domain of PA more likely to show observable associations with mental health [33, 68]. Additionally, accelerometer-based measurements may be more sensitive than other methods in capturing children’s activity, potentially contributing to higher observed effects [37]. Thus, accelerometer-based data collection may offer a useful complement in PA study, particularly when aiming to capture more detailed or nuanced activity [21, 24].

However, it is also evident that the confidence intervals in our meta-analysis are substantially wider compared to prior studies, this may be explained by methodological variability specific to accelerometer-based MVPA measurement [44]. Moreover, the results of the present study demonstrated that epoch length had an impact on the relationship between MVPA and depression. Specifically, an epoch with a duration of 15 s or less had a higher negative association between PA and depression (β = −0.32, *p* < 0.01) compared to an epoch exceeding 15 s (effect size = −0.08, *p* = 0.16). This may be because shorter epochs are less likely to miss important information about MVPA in children and adolescents, possibly due to the short bursts of activity that are typical for this age group [104, 105]. These short bursts of activity can be captured effectively by shorter epochs [106]. Consistent with our finding regarding the importance for epoch length, previous research has also demonstrated that epoch length affected the magnitude of the relationship between PA and other health outcomes, such as bone health, in children [25, 107, 108]. Moreover, in the subgroup analysis, although the heterogeneity of both epoch length groups was about 3% lower than the overall pooled heterogeneity, the heterogeneity in the group with epoch length greater than 15 s was 1.5% higher than that in the group with epoch length less than or equal to 15 s. This may be because longer epoch lengths are less stable in capturing the total amount of MVPA [43, 45], as there are large variations in the duration of activities and the intervals between high-intensity bouts in children [104, 105, 109]. Therefore, the present study suggest that using shorter epoch lengths may help reduce between-study heterogenerity [26, 110–113].

Accelerometer wear location may also be a sensitive measurement variable that affects the relationship between PA and mental health [103]. The present study found that PA captured by wrist-located accelerometer had a larger negative association (−0.29, 95% CI [−0.39, −0.19]) compared to the hip location (−0.10, 95% CI [−0.27, 0.06]). This may be explained by evidence from Hildebrand et al. [88], who found that wrist-worn monitors tend to record higher raw acceleration output than hip-worn monitors during many common activities. The wrist placement is particularly sensitive to upper-body and arm movements, which are often missed by hip-worn devices. Also, wrist-worn accelerometers may have higher compliance compared to hip-worn device [112]. However, despite these advantages, hip-worn may be more sensitive in capturing PA data. Whilst the relationship observed within this study are based on intensities established through published thresholds, there is an extensive discussion within the accelerometry literature relating to limitations of this classification method, which may have increased the heterogeneity among studies [114]. For example, short epoch lengths also have some limitations, such as data storage capacity issues [45]. Moreover, many intensity cut-points were developed based on longer epochs, such as 60 s, which may lead to biased PA estimates when these cut-points are applied alongside short epochs [42, 115]. Also, wrist placement also presents limitations, including the overestimation of movement during sedentary activities due to localized arm motion, reduced accuracy in detecting moderate-intensity activities (particularly in children), and inconsistencies arising from differences in device brands and data processing algorithms [88, 116]. This could also lead to the inclusion of misclassified intensities in the meta-analysis, thereby underestimating the true relationship. Therefore, research should address these limitations by incorporating standardized calibration protocols and exploring innovative activity metrics, which beyong the PA volume and intensity, and improve and standardise accelerometry methodologies [24, 26]. These will help to reduce measurement limitaion and the comparability of accelerometer-derived data across studies and age groups and decrease the heterogeneity between studies.

While the results of the present review support a beneficial association between PA and symptoms of depression and anxiety in children and adolescents, it is important to highlight that a negative association was observed for cross-sectional studies (*r* = −0.19, 95%CI [−0.34, −0.04], *p* = 0.01) but not for prospective studies (*r* = −0.17, 95%CI [−0.36, 0.02], *p* = 0.12). These results align with those by Poitras et al. [37], who reported findings from only one prospective study, and concluded no association between TPA, MVPA and depression. This lack of association might be attributed to the smaller number of prospective studies examined compared to the number of studies available. Moreover, it is important to highlight that the direction and magnitude of the effect size in both cross-sectional and prospective studies were strikingly similar, with only the confidence interval of the prospective studies crossing zero. This may be due to large sample size and multiple collection data points, which could increase variability and reduce the precision of the estimates [37]. One possible reason is that most studies measured PA only at baseline, lacking data on changes over time [36]. Additionally, some studies did not control for confounding factors, such as baseline mental health status, and different assessment tools were used to measure mental health at baseline and follow-up [74, 75]. 10 prospective studies examined in this review, 4 were categorised as low quality, which is 26% higher than the proportion of low-quality studies in the cross-sectional studies, where only 2 out of the fourteen studies were considered of poor quality. Only 2 studies [32, 65] reported sample size justification for the consistent implementation of outcome measures, and just half of the studies reported uniform selection criteria, repeated assessments of exposures over time and loss to follow-up. Future research requires more high-quality longitudinal studies to support the causal link. Additionally, experimental studies are crucial to establish causality and further validate these findings [117].

### Perspectives

Since the pooled effect size of the present meta-analysis is slight larger than that of previous studies (which typically included studies using self-reported questionnaires), this suggests that accelerometer-measured PA may be more stable and sensitive in capture children’ PA data compared to self-report PA. However, the wide confidence intervals of the effect size may indicate that accelerometer data collection and analysis methods have some influence on the magnitude of the association. We also identified two key methodological factors, epoch length and wear location that affected the observed relationship. In addition, results from the systematic review reported substantial variability across studies in how accelerometers were used for data collection and analysis. These factors limit comparability across studies and constrain further investigation into the relationship between PA and mental health. Therefore, this study suggests that future research should improve and standardise accelerometry methodologies and develop innovative PA metrics to reduce the impact of methodological heterogeneity. Finally, longitudinal studies, especially large-scale studies conducted over extended periods, remain very limited and should ensure consistency in the use of measurement tools and control of confounding variables.

This review has several limitations. First, the review was restricted to English-language publications and excluded clinical populations, which may have introduced selection and publication bias. Most large-scale studies using accelerometer-based measurements have been conducted in high-income countries, which may not be representative of children and adolescents globally, particularly limiting the understanding of the relationship between accelerometer-measured activity behaviours and mental health among those in low- and middle-income countries. Second, although the study pooled different effect sizes separately, such as partial *r* or odds ratio, to reduce potential pooling bias, this may limit statistical power and generalisability. Third, although differences in accelerometer data collection and analytical methods explained part of the between-study heterogeneity, substantial heterogeneity remained when combining subgroups with different methodological approaches in the moderator analyses. Due to limited subgroup sample sizes, further exploration was not feasible in the present study. Finally, the included studies used various questionnaires to assess depression and anxiety; however, we did not compare the moderating effects of different instruments, mainly because the sample size for some questionnaires was insufficient for pooling. Future studies could consider exploring the moderating role of mental health assessment tools.

## Conclusion

The study revealed a consistent small negative association between PA and symptoms of depression and anxiety in cross-sectional studies, while prospective studies, although aligned in direction and magnitude, did not reach statistical significance. More well-controlled prospective studies are essential to establish clearer insights and robust conclusions about the impact of PA on mental health in this demographic. However, high heterogeneity among studies suggests that factors such as measurement decisions contribute to variations, these factors, particularly epoch length and wear location, may serve as important moderators influencing the magnitude of the associations between PA and mental health. Further studies should improve and standardised accelerometry methodologies and developed innovative PA metrics are needed to better assess these relationships.

## Supplementary Information

Supplementary Material 1

## Data Availability

The data extraction tables used for this systematic review are available from the corresponding author upon reasonable request.

## Abbreviations

PA: Physical activity
MVPA: Moderate-to-vigorous physical activity
LPA: Light physical activity
VPA: Vigorous physical activity
TPA: Total physical activity
SB: Sedentary behaviour
BMI: Body mass index
OR: Odds ratio
CI: Confidence interval
SD: Standard deviation
CPM: Counts per minute
METs: Metabolic equivalents
AEE: Activity energy expenditure
SMFQ: Short mood and feelings questionnaire
SCAS-S: Spence children's anxiety scale—short version
SDQ: Strengths and difficulties questionnaire
CES-D: Center for epidemiologic studies depression scale
CDI: Children’s depression inventory
MFQ: Mood and feelings questionnaire
STAI-C: State-trait anxiety inventory for children
SCL-90: Symptom checklist-90
DASS-21: Depression anxiety stress scales-21
GHQ: General health questionnaire
BDI: Beck depression inventory
BSI: Brief symptom inventory
CAPA: Child and adolescent psychiatric assessment
PAPA: Preschool age psychiatric assessment
CIS-R: Clinical interview schedule-revised
BASC: Behaviour assessment system for children
PRISMA: Preferred reporting items for systematic reviews and meta-analyses
