# Supplementary material for: The association between accelerometer-measured physical activity and symptoms of depression and anxiety in children and adolescents: a systematic review and meta-analysis
Source: BMC Pediatr. 2025 Dec 28;26:96. doi: 10.1186/s12887-025-06420-y (PMC12882467; doi:10.1186/s12887-025-06420-y)
Supplement: Supplementary file 1 — Supplementary Material 1 [file 12887_2025_6420_MOESM1_ESM.docx]

**SUPPLEMENTARY MATERIAL LIST**

**Supplementary Material A**

PRISMA Checklist

Includes the PRISMA 2020 checklist with line numbers indicating where each item is addressed in the main manuscript.

**Supplementary Material B**

Search Strategy

Details the search strategy used across five databases (Medline, PsycINFO, Embase, Web of Science, and SPORTDiscus), including search terms, field codes, date of search, number of records retrieved, and search logic.

**Supplementary Material C**

Cohen’s Kappa Calculation Based on Actual Screening Decisions

Presents the inter-rater reliability (Cohen’s kappa) based on inclusion/exclusion decisions made by two independent reviewers, along with an interpretation of agreement level.

**Supplementary Material D**

Partial r Subgroup Analysis Table

Displays moderation analyses of the association between physical activity (PA) and mental health (partial r).

**Supplementary Material E**

Quality Assessment Table

Summarizes the quality ratings of included studies (cross-sectional and prospective), based on key methodological criteria. Studies are classified as “Good,” “Fair,” or “Poor,” with a brief synthesis of main quality limitations.

**Supplementary Material F**

Subgroup/Moderation Analysis Tables

Table F1: Subgroup analysis of moderate-to-vigorous PA (MVPA) and depression based on partial r values.

Table F2: Subgroup analysis of MVPA and depression based on odds ratios (OR).

Each table shows how different accelerometer processing variables (e.g., epoch size, wear location) influence the association strength.

**Supplementary Material G**

Sensitivity Analysis of Meta-analysis

G1 & G2: Leave-one-out analyses for the association between MVPA and depression/anxiety (partial r).

G3: Leave-one-out analysis for MVPA and depression (OR).

Figures demonstrate the robustness of results to the removal of individual studies.

Supplementary Material H

Publication Bias Analysis

H1: Funnel plot and trim-and-fill analysis for MVPA and depression (partial r).

H2: Funnel plot and sensitivity check for MVPA and anxiety (partial r).

H3: Funnel plot and trim-and-fill results for MVPA and depression (OR).

These analyses assess the risk of publication bias and confirm the stability of observed effects.

**Supplementary material A**

PRISMA check list

| Section and Topic | Item # | Checklist item | Location where item is reported (Line) |
| --- | --- | --- | --- |
| TITLE | | |  |
| Title | 1 | Identify the report as a systematic review. | 1-2 |
| ABSTRACT | | |  |
| Abstract | 2 | See the PRISMA 2020 for Abstracts checklist. | 4-30 |
| INTRODUCTION | | |  |
| Rationale | 3 | Describe the rationale for the review in the context of existing knowledge. | 50-132 |
| Objectives | 4 | Provide an explicit statement of the objective(s) or question(s) the review addresses. | 133-140 |
| METHODS | | |  |
| Eligibility criteria | 5 | Specify the inclusion and exclusion criteria for the review and how studies were grouped for the syntheses. | 156-166 |
| Information sources | 6 | Specify all databases, registers, websites, organisations, reference lists and other sources searched or consulted to identify studies. Specify the date when each source was last searched or consulted. | 142-144 |
| Search strategy | 7 | Present the full search strategies for all databases, registers and websites, including any filters and limits used. | 146-155 |
| Selection process | 8 | Specify the methods used to decide whether a study met the inclusion criteria of the review, including how many reviewers screened each record and each report retrieved, whether they worked independently, and if applicable, details of automation tools used in the process. | 177-188 |
| Data collection process | 9 | Specify the methods used to collect data from reports, including how many reviewers collected data from each report, whether they worked independently, any processes for obtaining or confirming data from study investigators, and if applicable, details of automation tools used in the process. | 168-176 |
| Data items | 10a | List and define all outcomes for which data were sought. Specify whether all results that were compatible with each outcome domain in each study were sought (e.g. for all measures, time points, analyses), and if not, the methods used to decide which results to collect. | 177-188 |
|  | 10b | List and define all other variables for which data were sought (e.g. participant and intervention characteristics, funding sources). Describe any assumptions made about any missing or unclear information. | 177-188 |
| Study risk of bias assessment | 11 | Specify the methods used to assess risk of bias in the included studies, including details of the tool(s) used, how many reviewers assessed each study and whether they worked independently, and if applicable, details of automation tools used in the process. | 247-250 |
| Effect measures | 12 | Specify for each outcome the effect measure(s) (e.g. risk ratio, mean difference) used in the synthesis or presentation of results. | 196-246 |
| Synthesis methods | 13a | Describe the processes used to decide which studies were eligible for each synthesis (e.g. tabulating the study intervention characteristics and comparing against the planned groups for each synthesis (item #5)). | 146-155 |
|  | 13b | Describe any methods required to prepare the data for presentation or synthesis, such as handling of missing summary statistics, or data conversions. | 205-221 |
|  | 13c | Describe any methods used to tabulate or visually display results of individual studies and syntheses. | 196-204 |
|  | 13d | Describe any methods used to synthesize results and provide a rationale for the choice(s). If meta-analysis was performed, describe the model(s), method(s) to identify the presence and extent of statistical heterogeneity, and software package(s) used. | 227-234 |
|  | 13e | Describe any methods used to explore possible causes of heterogeneity among study results (e.g. subgroup analysis, meta-regression). | 235-241 |
|  | 13f | Describe any sensitivity analyses conducted to assess robustness of the synthesized results. | 241-246 |
| Reporting bias assessment | 14 | Describe any methods used to assess risk of bias due to missing results in a synthesis (arising from reporting biases). | 247-250 |
| Certainty assessment | 15 | Describe any methods used to assess certainty (or confidence) in the body of evidence for an outcome. | 227-234 |
| RESULTS | | |  |
| Study selection | 16a | Describe the results of the search and selection process, from the number of records identified in the search to the number of studies included in the review, ideally using a flow diagram. | 252-262 |
|  | 16b | Cite studies that might appear to meet the inclusion criteria, but which were excluded, and explain why they were excluded. | 252-262 |
| Study characteristics | 17 | Cite each included study and present its characteristics. | 263-334 |
| Risk of bias in studies | 18 | Present assessments of risk of bias for each included study. | 335-351 |
| Results of individual studies | 19 | For all outcomes, present, for each study: (a) summary statistics for each group (where appropriate) and (b) an effect estimate and its precision (e.g. confidence/credible interval), ideally using structured tables or plots. | 353-434 |
| Results of syntheses | 20a | For each synthesis, briefly summarise the characteristics and risk of bias among contributing studies. | Supplementary Material |
|  | 20b | Present results of all statistical syntheses conducted. If meta-analysis was done, present for each the summary estimate and its precision (e.g. confidence/credible interval) and measures of statistical heterogeneity. If comparing groups, describe the direction of the effect. | 442-511 |
|  | 20c | Present results of all investigations of possible causes of heterogeneity among study results. | 484-511 |
|  | 20d | Present results of all sensitivity analyses conducted to assess the robustness of the synthesized results. | Supplementary Material |
| Reporting biases | 21 | Present assessments of risk of bias due to missing results (arising from reporting biases) for each synthesis assessed. | Supplementary Material |
| Certainty of evidence | 22 | Present assessments of certainty (or confidence) in the body of evidence for each outcome assessed. | 514-530 |
| DISCUSSION | | |  |
| Discussion | 23a | Provide a general interpretation of the results in the context of other evidence. | 533-630 |
|  | 23b | Discuss any limitations of the evidence included in the review. | 632-646 |
|  | 23c | Discuss any limitations of the review processes used. | 632-646 |
|  | 23d | Discuss implications of the results for practice, policy, and future research. | 648-655 |
| OTHER INFORMATION | | |  |
| Registration and protocol | 24a | Provide registration information for the review, including register name and registration number, or state that the review was not registered. | 32-33 |
|  | 24b | Indicate where the review protocol can be accessed, or state that a protocol was not prepared. | 131-133 |
|  | 24c | Describe and explain any amendments to information provided at registration or in the protocol. | 131-133 |
| Support | 25 | Describe sources of financial or non-financial support for the review, and the role of the funders or sponsors in the review. | 653-654 |
| Competing interests | 26 | Declare any competing interests of review authors. | 651-652 |
| Availability of data, code and other materials | 27 | Report which of the following are publicly available and where they can be found: template data collection forms; data extracted from included studies; data used for all analyses; analytic code; any other materials used in the review. | 648-650 |

**Supplementary material B**

Search strategy

| Table 1. Search per database | | | |
| --- | --- | --- | --- |
| Database searched | via | Years of coverage | Records |
| Medline ALL | Ovid | 1946 - Present | 1250 |
| PsycINFO | Ovid | 1806 - Present | 530 |
| Embase | Ovid | 1971 - Present | 2981 |
| Web of Science | Web of Knowledge | 1975 - Present | 515 |
| SPORTDiscus | EBSCOhost | 1975 - Present | 131 |
| Total | | | 5392 |

| Table 2A. Search Strategy in Medline (Ovid) | | |
| --- | --- | --- |
|  | Date of Search: 03_2022  Number of hits: 1250 | Field labels:  ti,ab.= titel & abstract  kf. = keywords  / = MeSH  exp/ = MeSH, exploded  adj = the terms are adjacent with no words in between in the order presented  adjx = adjacent within x words  * = truncation of word for alternate endings  $ = truncation of word for alternate endings |
|  | Term searched | Results |
| *Group 1* | *Participants (children and adolescents)* |  |
| 1 | Youth/ | 2166053 |
| 2 | Child/ | 1825373 |
| 3 | 1 or 2 | 3025540 |
| 4 | Adolescent/ | 2166053 |
| 5 | 3 or 4 | 3025540 |
| 6 | (Young adj people or Boy* or Girl* or Juvenile or Teen* or Teenager* or School adj age).ti,ab. | 17633 |
| 7 | 5 or 6 | 3029263 |
| Group 2 | Exposures (physical activity with accelerometer measurement) |  |
| 8 | Physical activity/ | 129840 |
| 9 | exercise/ | 129840 |
| 10 | 8 or 9 | 129840 |
| 11 | Sports/ | 32761 |
| 12 | 10 or 11 | 158070 |
| 13 | (Acute exercise* or Aerobic exercise* or Isometric exercise* or Fitness or Movement$ or Activi* or Motor activi* or Physical adj exertion or Habitual adj activi* or Sedentary adj Behavior or Fitness adj Trackers or Restraint adj Physical or Physical adj Endurance or Physical adj Fitness or Leisure adj Activit* or Human adj Activit* or Activity adj Cycle or Activation or Physical Education and Training or Physical adj Conditioning or Free adj living or physical adj effert or physical intensity or MVPA).ab,ti. | 5239 |
| 14 | 12 or 13 | 160291 |
| 15 | (bicycl$ or swim$ or walk$ or run$ or jog$).ab,ti. | 409213 |
| 16 | 14 or 15 | 551592 |
| 17 | (basketball OR baseball OR cycling OR football OR hockey OR icehockey OR fieldhockey OR jogging OR rugby OR running OR soccer OR softball OR tennis OR squash OR volleyball OR yoga OR fencing OR horse-riding OR horseback-riding OR wrestling OR judo OR karate OR jiu-jitsu OR taekwondo OR kung-fu OR martial-art* OR badminton).ab,ti. | 180910 |
| 18 | 16 or 17 | 651073 |
| 19 | Accelerometry.mp | 10157 |
| 20 | Actigraph.mp | 4438 |
| 21 | 19 or 20 | 12368 |
| 22 | Accelerometer.mp | 13057 |
| 23 | 21 or 22 | 19977 |
| 24 | (acceleromet* OR acceleration OR motion adj sensor OR accelerometer-measurement OR device-measured OR sensor-based measures OR motion adj sensor).af. | 598 |
| 25 | 23 or 24 | 20435 |
| 26 | (Count* or epoch adj size or cut-point or wear location or epoch* or non-wear or epoch length or threshold).af. | 278052 |
| 27 | 25 or 26 | 297430 |
| 28 | (activPAL or ActiGraph adj GT3X+ or Axivity adj AX3 adj tri-axial or GENEActiv).af. | 156 |
| 29 | 27 or 28 | 297450 |
| 30 | 18 AND 29 | 23793 |
| 31 | 7 AND 30 | 5012 |
| Group 3 | Outcomes (depression and anxiety) |  |
| 32 | Anxiety/ | 96451 |
| 33 | Depression/ | 138798 |
| 34 | 32 or 33 | 201115 |
| 35 | (Depression adj disrupti* OR Depression adj problem* OR Depression adj disorder* OR Depression adj disturb*).ab,ti. | 31 |
| 36 | 34 or 35 | 201131 |
| 37 | (Mental adj health or Mental adj hygiene or low adj mood or emotion or Anhedonia or Anxiety adj neuro* or Cortical adj Spreading or anxiety adj state or attention adj3 deficit* or anger or trait adj anxiety or fear or antisocial* or anxiolytic or Stress).ab,ti. | 944030 |
| 38 | 36 or 37 | 1103380 |
| 39 | 31 and 38 | 1315 |
| 40 | Limit 39 to English language | 1250 |

| Table 2B. Search Strategy in PsycINFO (Ovid) | | |
| --- | --- | --- |
|  | Date of Search: 24/03/2022  Number of hits: 530 | Field labels:  ti,ab.= titel & abstract  kf. = keywords  / = MeSH  exp/ = MeSH, exploded  adj = the terms are adjacent with no words in between in the order presented  adjx = adjacent within x words  * = truncation of word for alternate endings  $ = truncation of word for alternate endings |
|  | Term searched | Results |
| *Group 1* | *Participants (children and adolescents)* |  |
| 1 | Youth/ab,ti. | 99060 |
| 2 | Child/ab,ti. | 267987 |
| 3 | 1 or 2 | 351492 |
| 4 | Adolescent/ab,ti. | 117129 |
| 5 | 3 or 4 | 429548 |
| 6 | (Young adj people or Boy* or Girl* or Juvenile or Teen* or Teenager* or School adj age).ti,ab. | 14425 |
| 7 | 5 or 6 | 439259 |
| Group 2 | Exposures (physical activity) |  |
| 8 | Physical activity/ | 23362 |
| 9 | exercise/ | 25495 |
| 10 | 8 or 9 | 43500 |
| 11 | Sports/ | 21221 |
| 12 | 10 or 11 | 62766 |
| 13 | (Acute exercise* or Aerobic exercise* or Isometric exercise* or Fitness or Movement$ or Activi* or Motor activi* or Physical adj exertion or Habitual adj activi* or Sedentary adj Behavior or Fitness adj Trackers or Restraint adj Physical or Physical adj Endurance or Physical adj Fitness or Leisure adj Activit* or Human adj Activit* or Activity adj Cycle or Activation or Physical Education and Training or Physical adj Conditioning or Free adj living or physical adj effert or physical intensity or MVPA).ab,ti. | 2164 |
| 14 | 12 or 13 | 63412 |
| 15 | (bicycl$ or swim$ or walk$ or run$ or jog$).ab,ti. | 88575 |
| 16 | 14 or 15 | 144507 |
| 17 | (basketball OR baseball OR cycling OR football OR hockey OR icehockey OR fieldhockey OR jogging OR rugby OR running OR soccer OR softball OR tennis OR squash OR volleyball OR yoga OR fencing OR horse-riding OR horseback-riding OR wrestling OR judo OR karate OR jiu-jitsu OR taekwondo OR kung-fu OR martial-art* OR badminton).ab,ti. | 41704 |
| 18 | 16 or 17 | 161678 |
| 19 | Accelerometry.mp | 1706 |
| 20 | Actigraph.mp | 1113 |
| 21 | 19 or 20 | 2604 |
| 22 | Accelerometer.mp | 2385 |
| 23 | 21 or 22 | 4105 |
| 24 | (acceleromet* OR acceleration OR motion adj sensor OR accelerometer-measurement OR device-measured OR sensor-based measures OR motion adj sensor).af. | 368 |
| 25 | 23 or 24 | 4404 |
| 26 | (Count* or epoch adj size or cut-point or wear location or epoch* or non-wear or epoch length or threshold).af. | 114630 |
| 27 | 25 or 26 | 118618 |
| 28 | (activPAL or ActiGraph adj GT3X+ or Axivity adj AX3 adj tri-axial or GENEActiv).af. | 42 |
| 29 | 27 or 28 | 118628 |
| 30 | 18 AND 29 | 7289 |
| 31 | 7 AND 30 | 2245 |
| Group 3 | Outcomes (depression and anxiety) |  |
| 32 | Anxiety/ | 69700 |
| 33 | Depression/ | 26482 |
| 34 | 32 or 33 | 91175 |
| 35 | (Depression adj disrupti* OR Depression adj problem* OR Depression adj disorder* OR Depression adj disturb*).ab,ti. | 17 |
| 36 | 34 or 35 | 91191 |
| 37 | (Mental adj health or Mental adj hygiene or low adj mood or emotion or Anhedonia or Anxiety adj neuro* or Cortical adj Spreading or anxiety adj state or attention adj3 deficit* or anger or trait adj anxiety or fear or antisocial* or anxiolytic or Stress).ab,ti. | 320623 |
| 38 | 36 or 37 | 385654 |
| 39 | 31 and 38 | 548 |
| 40 | Limit 39 to English language | 530 |

| Table 2C. Search Strategy in Embase (Ovid) | | |
| --- | --- | --- |
|  | Date of Search: 24/03/2022  Number of hits: 2981 | Field labels:  ti,ab.= titel & abstract  kf. = keywords  / = MeSH  exp/ = MeSH, exploded  adj = the terms are adjacent with no words in between in the order presented  adjx = adjacent within x words  * = truncation of word for alternate endings  $ = truncation of word for alternate endings |
|  | Term searched | Results |
| *Group 1* | *Participants (children and adolescents)* |  |
| 1 | Youth/.ab,ti. | 94503 |
| 2 | Child/.ab,ti. | 469952 |
| 3 | 1 or 2 | 551352 |
| 4 | Adolescent/.ab,ti. | 173120 |
| 5 | 3 or 4 | 681863 |
| 6 | (Young adj people or Boy* or Girl* or Juvenile or Teen* or Teenager* or School adj age).ti,ab. | 22907 |
| 7 | 5 or 6 | 698902 |
| Group 2 | Exposures (physical activity) |  |
| 8 | Physical activity/ | 185268 |
| 9 | exercise/ | 308304 |
| 10 | 8 or 9 | 461964 |
| 11 | Sports/ | 49370 |
| 12 | 10 or 11 | 498604 |
| 13 | (Acute exercise* or Aerobic exercise* or Isometric exercise* or Fitness or Movement$ or Activi* or Motor activi* or Physical adj exertion or Habitual adj activi* or Sedentary adj Behavior or Fitness adj Trackers or Restraint adj Physical or Physical adj Endurance or Physical adj Fitness or Leisure adj Activit* or Human adj Activit* or Activity adj Cycle or Activation or Physical Education and Training or Physical adj Conditioning or Free adj living or physical adj effert or physical intensity or MVPA).ab,ti. | 6251 |
| 14 | 12 or 13 | 499864 |
| 15 | (bicycl$ or swim$ or walk$ or run$ or jog$).ab,ti. | 550346 |
| 16 | 14 or 15 | 989213 |
| 17 | (basketball OR baseball OR cycling OR football OR hockey OR icehockey OR fieldhockey OR jogging OR rugby OR running OR soccer OR softball OR tennis OR squash OR volleyball OR yoga OR fencing OR horse-riding OR horseback-riding OR wrestling OR judo OR karate OR jiu-jitsu OR taekwondo OR kung-fu OR martial-art* OR badminton).ab,ti. | 211577 |
| 18 | 16 or 17 | 1090795 |
| 19 | Accelerometry.mp | 10919 |
| 20 | Actigraph.mp | 6381 |
| 21 | 19 or 20 | 15838 |
| 22 | Accelerometer.mp | 21978 |
| 23 | 21 or 22 | 30326 |
| 24 | (acceleromet* OR acceleration OR motion adj sensor OR accelerometer-measurement OR device-measured OR sensor-based measures OR motion adj sensor).af. | 5137 |
| 25 | 23 or 24 | 33187 |
| 26 | (Count* or epoch adj size or cut-point or wear location or epoch* or non-wear or epoch length or threshold).af. | 365398 |
| 27 | 25 or 26 | 396867 |
| 28 | (activPAL or ActiGraph adj GT3X+ or Axivity adj AX3 adj tri-axial or GENEActiv).af. | 273 |
| 29 | 27 or 28 | 396887 |
| 30 | 18 AND 29 | 39839 |
| 31 | 7 AND 30 | 7341 |
| Group 3 | Outcomes (depression and anxiety) |  |
| 32 | Anxiety/ | 241287 |
| 33 | Depression/ | 415087 |
| 34 | 32 or 33 | 573288 |
| 35 | (Depression adj disrupti* OR Depression adj problem* OR Depression adj disorder* OR Depression adj disturb*).ab,ti. | 49 |
| 36 | 34 or 35 | 573309 |
| 37 | (Mental adj health or Mental adj hygiene or low adj mood or emotion or Anhedonia or Anxiety adj neuro* or Cortical adj Spreading or anxiety adj state or attention adj3 deficit* or anger or trait adj anxiety or fear or antisocial* or anxiolytic or Stress).ab,ti. | 1183039 |
| 38 | 36 or 37 | 1656239 |
| 39 | 31 and 38 | 3104 |
| 40 | Limit 39 to English language | 2981 |

| Table 2D. Search Strategy in Web of Science | | |
| --- | --- | --- |
|  | Date of Search 24/03/2022  Number of hits: | Field labels:  TS= TITILE |
|  | Term searched | Results |
| TS | Youth* or Child* or Adolescent* or “Young people” or Boy* or Girl* or Juvenile or Teen* or Teenager or “School age” | 2,759,495 |
| AND | (“Physical activity” or exercise* or Sport* or “Acute exercise*” or “Aerobic exercise*” or “Isometric exercise*” or Fitness or Movement$ or Activi* or “Physical exertion” or “Habitual activi*” or “Sedentary Behavior” or “Fitness Trackers” or “Restraint Physical” or “Physical Endurance” or “Physical Fitness” or “Leisure Activit*” or “Human Activit*” or “Activity Cycle” or Activation or “Physical Education” and Training or “Physical Conditioning” or “Free living” or “physical effert” or “physical intensity” or MVPA or bicycle* or swim or walk or run or jog or basketball OR baseball OR cycling OR football OR hockey OR icehockey OR fieldhockey OR jogging OR rugby OR running OR soccer OR softball OR tennis OR squash OR volleyball OR yoga OR fencing OR horse-riding OR horseback-riding OR wrestling OR judo OR karate OR jiu-jitsu OR taekwondo OR kung-fu OR martial-art* OR badminton) and (Accelerometry or Actigraph or Accelerometer or acceleromet* OR acceleration OR “motion sensor” OR accelerometer-measurement OR device-measured OR sensor-based measures OR “motion sensor”) or (activPAL or “ActiGraph GT3X+” or “Axivity AX3 tri-axial” or GENEActiv) | 7689 |
|  |  |  |
| AND | Anxiety* or Depression* or “Depression disrupti*” OR “Depression problem*” OR “Depression disorder*” OR “Depression disturb*” or “Mental health” or “Mental hygiene” or “low mood” or emotion or Anhedonia or “Anxiety neuro*” or “Cortical Spreading” or “anxiety state” or “attention deficit*” or anger or “trait anxiety” or fear or antisocial* or anxiolytic or Stress | 515 |
|  |  |  |
| Table 2E. Search Strategy in SPORTDiscus (EBSCOhost) | | |
|  | Date of Search 24/03/2022  Number of hits: 390 | Field labels:  TX= All Text |
|  | Term searched | Results |
| TX | Youth* or Child* or Adolescent* or “Young people” or Boy* or Girl* or Juvenile or Teen* or Teenager or “School age” | 211822 |
| AND | “Physical activity” or exercise* or Sport* or “Acute exercise*” or “Aerobic exercise*” or “Isometric exercise*” or Fitness or Movement$ or Activi* or “Physical exertion” or “Habitual activi*” or “Sedentary Behavior” or “Fitness Trackers” or “Restraint Physical” or “Physical Endurance” or “Physical Fitness” or “Leisure Activit*” or “Human Activit*” or “Activity Cycle” or Activation or “Physical Education” and Training or “Physical Conditioning” or “Free living” or “physical effert” or “physical intensity” or MVPA or bicycle* or swim or walk or run or jog or basketball OR baseball OR cycling OR football OR hockey OR icehockey OR fieldhockey OR jogging OR rugby OR running OR soccer OR softball OR tennis OR squash OR volleyball OR yoga OR fencing OR horse-riding OR horseback-riding OR wrestling OR judo OR karate OR jiu-jitsu OR taekwondo OR kung-fu OR martial-art* OR badminton AND Accelerometry or Actigraph or Accelerometer or acceleromet* OR acceleration OR “motion sensor” OR accelerometer-measurement OR device-measured OR sensor-based measures OR “motion sensor” or activPAL or “ActiGraph GT3X+” or “Axivity AX3 tri-axial” or GENEActiv | 2893 |
|  |  |  |
| AND | Anxiety* or Depression* or “Depression disrupti*” OR “Depression problem*” OR “Depression disorder*” OR “Depression disturb*” or “Mental health” or “Mental hygiene” or “low mood” or emotion or Anhedonia or “Anxiety neuro*” or “Cortical Spreading” or “anxiety state” or “attention deficit*” or anger or “trait anxiety” or fear or antisocial* or anxiolytic or Stress | 113 |
|  |  |  |

**Supplementary material C**

# Cohen’s Kappa Calculation Based on Actual Screening Decisions

To address the reviewer’s request regarding the inter-rater reliability of the study selection process, we calculated Cohen’s kappa using actual inclusion and exclusion decisions made by two reviewers (L and Y). A total of 4,260 records were screened. L included 70 records in total. Among these, 41 were excluded by Y. Meanwhile, 46 records were included by Y but excluded by L.

## Agreement Table

|  | Y: include | Y: exclude | Total |
| --- | --- | --- | --- |
| L: include | a = 29 | b = 41 | 70 |
| L: exclude | c = 46 | d = 4144 | 4190 |
| Total | 75 | 4185 | 4260 |

## Kappa Calculation

Observed agreement (Po) = (a + d) / N = (29 + 4144) / 4260 = 0.9796

Expected agreement (Pe) = ((a + b)/N × (a + c)/N) + ((c + d)/N × (b + d)/N) = 0.9665

Cohen’s kappa = (Po - Pe) / (1 - Pe) = 0.3896

## Interpretation

The computed kappa value is κ = 0.39, indicating fair agreement, according to Landis and Koch (1977). This level of agreement supports the reliability of the screening process.

**Supplementary material D**

Partial r covering for subgroup analysis effect

| Data processing of accelerometer | Cross-sectional studies | | Prospective studies | |
| --- | --- | --- | --- | --- |
| Epoch size | Partial r in MVPA  n=8 |  | Partial r in MVPA  n=6 |  |
| ≤ 15s | k=4  Zahl (2017) (6y) = -0.4471 (10s)  Zahl (2017) (8y) = -0.2972  Zahl (2017) (10y) = -0.463 |  | k=4  Zahl (2017)6_8 = -0.23 (10s)  Zahl (2017)6_10 = -0.23  Zahl (2017)8_10 = -0.20  Kracht (2023) = -0.50 (15s) |  |
| > 15s | k=4  Farren (2018) = -0.34 (60s)  Booth (2023) = -0.04 (60s)  Hagemann (2021) = -0.0009 (60s)  Parfitt (2009) = -0.037 (60s) |  | k=2  Booth (2023) = 0.0025 (60s)  Hume (2011) = -0.015 (60s) |  |
| Valid day | n=9 |  | n=6 |  |
| ≤ 3 | k=5  Zahl (2017) (6y) = -0.4471 (3d)  Zahl (2017) (8y) = -0.2972  Zahl (2017) (10y) = -0.463  Ghorbani (2021) = -0.29 (3d)  Booth (2023) = -0.04 (3d) |  | k=4  Zahl (2017)6_8 = -0.23 (3d)  Zahl (2017)6_10 = -0.23  Zahl (2017)8_10 = -0.20  Booth (2023) = 0.0025 (3d) |  |
| > 3 | k=4  Farren (2018) = -0.34 (4d)  Hagemann (2021) = -0.0009 (4d)  Parfitt (2009) = -0.037 (4d) |  | k=2  Booth (2023) = 0.0025 (4d)  Hume (2011) = -0.015 (4d) |  |
| Valid hours | n=8 |  | n=5 |  |
| ≤ 8 | k=4  Zahl (2017) (6y) = -0.4471 (8h)  Zahl (2017) (8y) = -0.2972  Zahl (2017) (10y) = -0.463  Ghorbani (2021) = -0.29 (8h) |  | k=3  Zahl (2017)6_8 = -0.23 (8h)  Zahl (2017)6_10 = -0.23  Zahl (2017)8_10 = -0.20 |  |
| > 8 | k=4  Booth (2023) = -0.04 (10h)  Parfitt (2009) = -0.037 (10h)  Farren (2018) = -0.34 (13h) |  | k=2  Hume (2011) = -0.015 (10h)  Kracht et al (2023) = -0.50 (10h) |  |
| Wear location | n=9 |  | n=6 |  |
| Hip | k= 3  Booth (2023) = -0.04  Parfitt (2009) = -0.037  Hagemann (2021) = -0.0009 |  | k=3  Hume (2011) = -0.015  Kracht et al (2023) = -0.50  Booth (2023) = 0.0025 |  |
| Wrist | k= 6  Farren (2018) = -0.34  Ghorbani (2021) = -0.29  Zahl (2017) (6y) = -0.4471  Zahl (2017) (8y) = -0.2972  Zahl (2017) (10y) = -0.463 |  | k=3  Zahl (2017)6_8 = -0.23  Zahl (2017)6_10 = -0.23  Zahl (2017)8_10 = -0.20 |  |
| Weekend included | n=9 |  | n=6 |  |
| Yes | k=6  Zahl (2017) (6y) = -0.4471  Zahl (2017) (8y) = -0.2972  Zahl (2017) (10y) = -0.463  Booth (2023) = -0.04  Parfitt (2009) = -0.037 |  | k=6  Zahl (2017)6_8 = -0.23  Zahl (2017)6_10 = -0.23  Zahl (2017)8_10 = -0.20  Hume (2011) = -0.015  Kracht et al (2023) = -0.50  Booth (2023) = 0.0025 |  |
| No | k=3  Farren (2018) = -0.34  Ghorbani (2021) = -0.29  Hagemann (2021) = -0.0009 |  | k=0 |  |

**Supplementary material E**

Quality Assessment Table

| Reference | 1. Was the research question or objective in this paper clearly stated? | 2. Was the study population clearly specified and defined? | 3. Was the participation rate of eligible persons at least 50%? | 4. Were all the subjects selected or recruited from the same or similar populations (including the same time period)? Were inclusion and exclusion criteria for being in the study prespecified and applied uniformly to all participants? | 5. Was a sample size justification, power description, or variance and effect estimates provided? | 6. For the analyses in this paper, were the exposure(s) of interest measured prior to the outcome(s) being measured? | 7. Was the timeframe sufficient so that one could reasonably expect to see an association between exposure and outcome if it existed? | 8. For exposures that can vary in amount or level, did the study examine different levels of the exposure as related to the outcome (e.g., categories of exposure, or exposure measured as continuous variable)? | 9. Were the exposure measures (independent variables) clearly defined, valid, reliable, and implemented consistently across all study participants? | 10. Was the exposure(s) assessed more than once over time? | 11. Were the outcome measures (dependent variables) clearly defined, valid, reliable, and implemented consistently across all study participants? | 12. Were the outcome assessors blinded to the exposure status of participants? | 13. Was loss to follow-up after baseline 20% or less? | 14. Were key potential confounding variables measured and adjusted statistically for their impact on the relationship between exposure(s) and outcome(s)? | QAULITY |
| --- | --- | --- | --- | --- | --- | --- | --- | --- | --- | --- | --- | --- | --- | --- | --- |
| **Cross-sectional study** | | | | | | | | | | | | | | | |
| Johnson et al (2008) | Y | Y | Y | N | N | NA | NA | Y | Y | NA | Y | N | Y | Y | FAIR |
| Parfitt et al (2009) | Y | Y | Y | N | N | NA | NA | Y | Y | Y | N | N | N | Y | FAIR |
| Wiles et al (2012) | Y | Y | Y | N | N | NA | NA | Y | Y | NA | N | N | N | Y | FAIR |
| Martikainen et al (2012) | Y | Y | Y | Y | N | NA | NA | N | Y | NA | N | N | N | Y | FAIR |
| Farren et al (2018) | Y | Y | N | N | N | NA | NA | Y | Y | NA | Y | N | Y | Y | FAIR |
| Hrafnkelsdottir et al (2018) | Y | Y | Y | N | N | NA | NA | Y | Y | NA | N | N | N | Y | FAIR |
| Hagemann et al (2021) | Y | Y | N | Y | Y | NA | NA | Y | Y | NA | Y | N | N | Y | FAIR |
| Ghorbani et al (2021) | Y | Y | Y | N | N | NA | NA | Y | Y | NA | N | N | Y | Y | FAIR |
| Dumuid et al (2021) | Y | Y | N | N | N | NA | NA | Y | Y | NA | N | N | N | Y | POOR |
| Fairclough et al (2021) | Y | Y | Y | Y | N | NA | NA | Y | Y | NA | Y | N | Y | Y | FAIR |
| da Costa et al (2022a) | Y | Y | Y | Y | N | NA | NA | Y | Y | NA | Y | N | N | Y | FAIR |
| Kjellenberg et al (2022) | Y | Y | Y | N | N | NA | NA | Y | Y | NA | Y | N | Y | Y | FAIR |
| de Faria et al (2022) | Y | Y | Y | Y | Y | NA | NA | Y | Y | NA | Y | N | Y | Y | GOOD |
| da Costa et al (2022b) | Y | Y | Y | N | N | NA | NA | Y | Y | NA | N | N | N | Y | POOR |
| Fairclough et al. (2023) | Y | Y | Y | N | N | NA | NA | Y | Y | NA | Y | N | Y | Y | FAIR |
| **Prospective study** | | | | | | | | | | | | | | | |
| Van Dijk et al (2016) | Y | Y | Y | N | N | Y | N | N | Y | Y | N | N | N | Y | POOR |
| Zahl et al (2017) | Y | Y | Y | Y | N | Y | Y | Y | Y | Y | N | N | Y | N | FAIR |
| Ahn et al (2018) | Y | Y | N | Y | N | Y | Y | Y | Y | N | N | N | Y | Y | FAIR |
| Bell et al (2019) | Y | Y | Y | N | N | Y | Y | Y | Y | N | Y | N | Y | Y | FAIR |
| Hamer et al (2020) | Y | Y | N | Y | N | Y | Y | Y | Y | Y | Y | N | Y | Y | FAIR |
| Kandola et al (2020b) | Y | Y | N | N | N | Y | Y | Y | Y | Y | N | N | N | Y | POOR |
| Kandola et al (2020a) | Y | Y | N | N | N | Y | Y | Y | Y | Y | N | N | N | Y | POOR |
| Slykerman et al (2020) | Y | Y | Y | Y | N | Y | Y | Y | N | N | N | N | N | Y | POOR |
| Booth et al (2023) | Y | Y | Y | Y | N | Y | Y | Y | Y | N | Y | N | N | Y | FAIR |
| Hume et al (2011) | Y | Y | N | Y | N | Y | Y | Y | N | Y | N | N | N | Y | FAIR |
| Nyberg et al (2023) | Y | Y | Y | N | N | Y | Y | Y | Y | Y | Y | N | N | Y | FAIR |
| Kracht et al (2023) | Y | Y | Y | Y | N | Y | Y | Y | Y | Y | Y | N | Y | Y | FAIR |
| Yang al (2023) | Y | Y | Y | Y | N | Y | Y | Y | Y | N | Y | N | Y | Y | FAIR |
| Monteagudo et al (2023) | Y | Y | N | Y | Y | Y | Y | Y | Y | Y | N | Y | N | N | FAIR |
| Haapala et al. (2025) | Y | Y | Y | Y | NR | Y | Y | Y | Y | Y | Y | NR | Y | Y | GOOD |

In assessing the quality of fourteen cross-sectional studies, one study was categorized as “Good” (de Faria et al., 2022), 13 as "Fair” (da Costa, Chaput, et al., 2022; Fairclough et al., 2023; Fairclough et al., 2021; Farren et al., 2018; Ghorbani et al., 2021; Hagemann et al., 2021; Hrafnkelsdottir et al., 2018; Johnson et al., 2008; Kjellenberg et al., 2022; Martikainen et al., 2012; Parfitt, Pavey, & Rowlands, 2009; Wiles et al., 2012) and two (da Costa, Bruner, et al., 2022; Dumuid et al., 2021) as "Poor" quality .

In the quality assessment of 15 prospective studies, 1 study was categorized as “Good” (Haapala et al., 2025); 10 studies (Ahn et al., 2018; Bell et al., 2019; Booth et al., 2023; Hamer et al., 2020; Hume. et al., 2011; Kracht, Pochana, & Staiano, 2023; Monteagudo et al., 2023; Nyberg et al., 2023; Van Dijk et al., 2016; Yang, Corpeleijn, & Hartman., 2023; Zahl, Steinsbekk, & Wichstrom, 2017) were rated as “Fair” and 4 studies (Clare Hume, 2011; Kandola et al., 2020; Kandola. et al., 2020; Slykerman et al., 2020) as “Poor”. The main quality issues were that no studies reported on the blinding of outcome assessors, only two studies (de Faria et al., 2022; Hagemann et al., 2021) reported sample size justification the consistent implementation of outcome measures, and just half of the studies reported uniform selection criteria, repeated assessments of exposures over time and loss to follow-up.

**Supplementary material F**

Table F1 accelerometer data processing moderation analysis on the relationship between MVPA and depression for partial r results

| Moderation variables | | Sample size | partial r | *I^2^* | Weight | Chi2 |
| --- | --- | --- | --- | --- | --- | --- |
| Epoch size (≤ 15 second)  Epoch size (> 15 second) | | 4  5 | -0.32 (-0.49, -0.15)  -0.09(-0.22, 0.04) | 93 %  96.33% | 45.22 %  56.78 % | 7.40 (*p*= 0.01) |
| Valid day (≤ 3days)  Valid day (>3days) | | 5  6 | -0.19 (-0.34, -0.03)  -0.17 (-0.35, 0.00) | 98.22%  93.15% | 57.69 %  51.31 % | 0.00 (*p* = 0.92) |
| Valid hour (≤ 8 hours)  Valid hour (> 8 hours) | | 3  6 | -0.31 (-0.43, -0.19)  -0.17 (-0.35, 0.00) | 81.87 %  98.14 % | 34.58 %  65.42 % | 1.01 (*p* = 0.21) |
|  | |  |  |  |  |  |
| Wear location (wrist)  Wear location (hip) | | 5  6 | -0.29 ( -0.39, -0.19)  -0.10 (-0.27, 0.06) | 76.33 %  98.18 % | 45.13 %  44.87 % | 3.48 (*p* = 0.06) |
| Weekends include (yes)  Weekends include (no) | | 2  8 | -0.29 (-0.75, 0.17)  -0.16 (-0.28, -0.04) | 90.87 %  97.14 % | 17.65 %  82.36 % | 0.28 (*p* = 0.60) |
|  |  | |  |  |  |  |

Table F1 illustrates the moderating effects of accelerometer data processing variables on the association between MVPA and depression. The analysis considers various data processing parameters, such as epoch size, the number of valid days and hours of accelerometer wear, wear location, and whether weekends are included in the data. The results indicate that the epoch size of ≤ 15 seconds and the wear location on the wrist are significant moderators in the relationship between MVPA and depression. A smaller epoch size has shown a significant moderating effect, suggesting that finer temporal resolutions in accelerometer data may reveal stronger associations with depression. Similarly, the wrist as a wear location for the accelerometer is another significant moderator. Conversely, other processing variables such as a larger epoch size (> 15 seconds), the number of valid days (whether ≤3 days or >3 days), the number of valid wear hours (whether ≤8 hours or >8 hours), and the inclusion of weekends did not exhibit a significant moderating effect. This suggests that these aspects of accelerometer data processing do not substantially alter the observed association between MVPA and depression. In summary, the table presents evidence that specific accelerometer data processing decisions, particularly related to epoch size and wear location, can significantly influence the observed correlation between physical activity levels and depression.

Subgroup analysis table of meta-analysis

Table F2 Accelerometer data processing moderation analysis on the association between MVPA and depression for OR results

| Moderation variables | Sample size | Effect size | *I^2^* | Weight | Chi2 |
| --- | --- | --- | --- | --- | --- |
| Epoch size (≤ 15 second)  Epoch size (> 15 second) | 1  4 | 1.04 (0.86, 1.17)  0.97 (0.95, 0.99) | 0  21.78 % | 2.66%  97.37 % | 0.13 (*p*= 0.71) |
| Valid day (≤ 3days)  Valid day (>3days) | 2  3 | 0.97 (0.95, 0.99)  0.97 (0.95, 0.99) | 35.92 %  0 % | 92.45 %  7.58 % | 0.12 (*p* = 0.73) |
| Wear location (wrist)  Wear location (hip) | 2  3 | 0.81 (0.28, 2.38)  0.97 (0.95, 0.99) | 52.01 %  19.18 % | 4.94 %  95.06 % | 0.10 (*p* = 0.74) |
| Weekends included (yes)  Weekends included (no) | 2  3 | 0.82 (0.36, 1.87)  0.95 (0.88, 1.02) | 45.90 %  90.23 % | 4.94 %  95.06 % | 0.12 (*p* = 0.73) |

Table F2 presents a moderation analysis that examines how various accelerometer data processing variables affect the association between MVPA and depression, reported in terms of OR. The moderation variables investigated include epoch size, the number of valid days and hours of accelerometer wear, wear location, and whether weekends were included in the analysis. None of these variables showed statistically significant moderation effects on the relationship between MVPA and depression.

**Supplementary material G**

Sensitivity analysis of meta-analysis

G1. leaves one out of the association between MVPA and depression for partial *r*


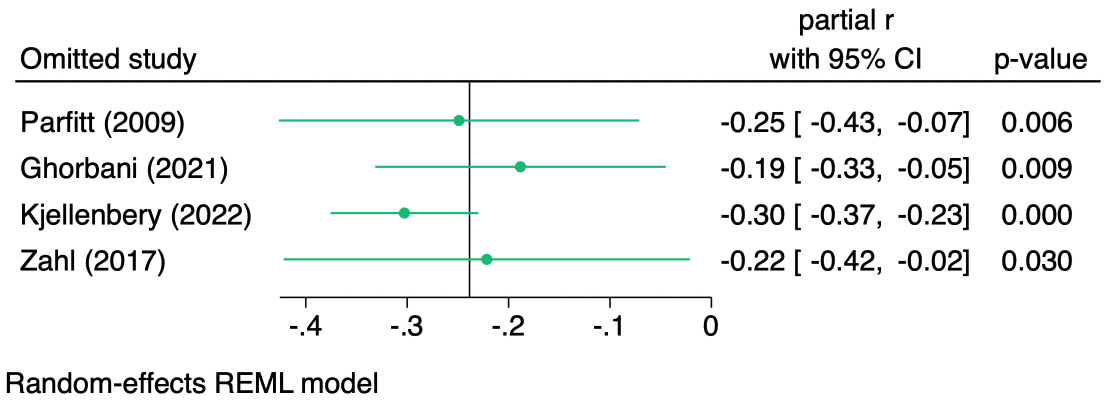


G2. leaves one out of the association between MVPA and anxiety for partial *r*

Figure G1 and G2 show that neither the magnitude or significance of the overall partial r change as a result of the leave-one-out sensitivity analysis (Depression: partial r = -0.14 to -0.19, p=0.003 to 0.001, Anxiety: -0.19 to -0.30, p=0.03 to <0.001)


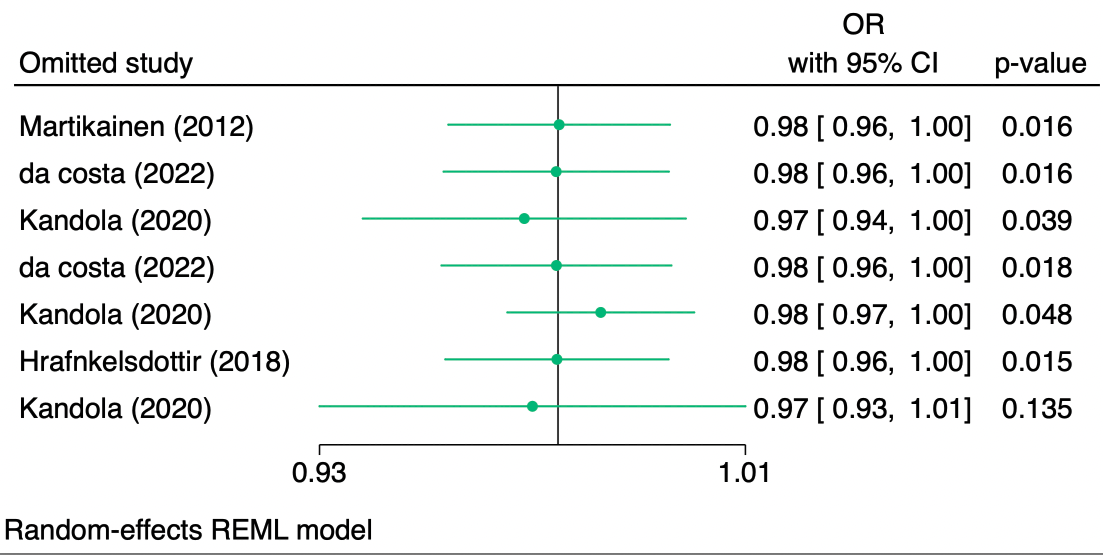


G3. leaves one out of the association between PA and depression for OR result.

Figure F3 shows that the magnitude of the overall odds ratio did not change as a result of the leave-one-out sensitivity analysis (Depression: OR=0.97 to 0.98) and all-but-one remained statistically significant (p=0.015 to 0.048). Only the removal of the 'Kandola 2020' study led to a non-sig overall association (OR=0.97, p-0.135).

**Supplementary material H**

Publication bias analysis of meta-analysis

H1. The publication bias of the association between MVPA and depression

H1. Funnel plot of the association between MVPA and depression

Figure H1 shows that there is no apparent publication bias in the meta-analysis examining the association between MVPA (moderate to vigorous physical activity) and depression. The absence of imputed studies in the trim-and-fill analysis suggest that the observed effect size remains unchanged ('partial r' = -0.184) with a 95% confidence interval ranging from -0.305 to -0.006, indicating a small but negative association between MVPA and depression.

H 2. The publication bias of the association between MVPA and anxiety

H2 Funnel plot of the association between MVPA and anxiety

Figure H2 shows that there is no apparent publication bias in the meta-analysis examining the association between MVPA (moderate to vigorous physical activity) and depression. The leave-one-out sensitivity analysis showed that the effect size remained relatively stable when any single study was omitted (ranging from -0.188 to -0.303), with a consistent negative direction. The combined effect size remained statistically significant in all cases (p-values < 0.05). These results indicate that the findings are not sensitive to any individual study, suggesting that the overall conclusion is robust and reliable.

H3 The publication bias of the association between MVPA and depression for OR results


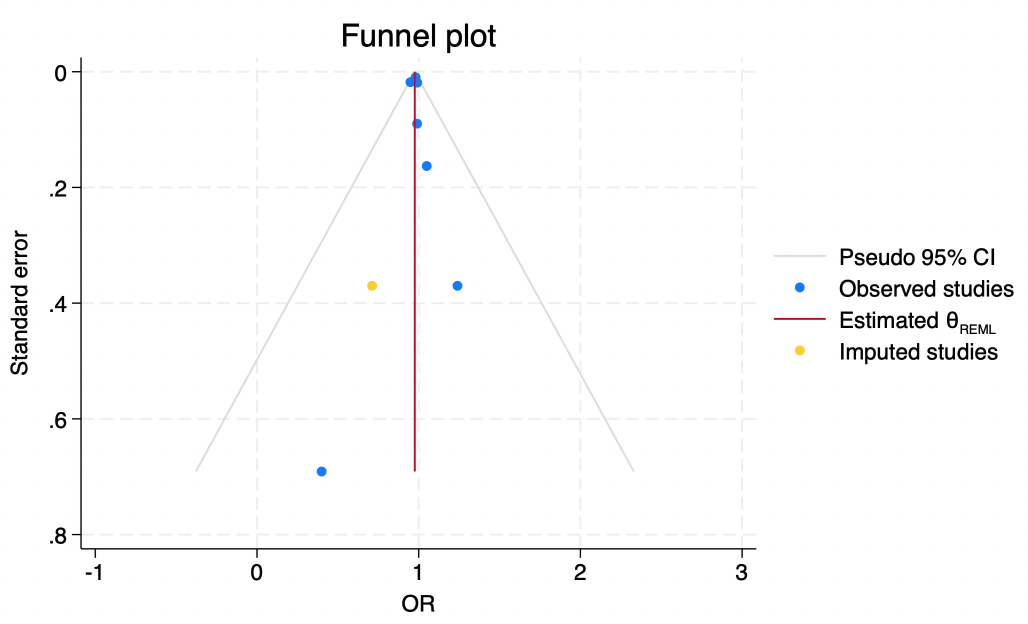


H3 Publication bias of the association between PA and depression

Figure H3 shows that shows that there is no apparent publication bias in the meta-analysis examining the association between PA and depression. the observed effect size indicates a slight, non-significant reduction in the odds of depression associated with MVPA (OR = 0.976, 95% CI: 0.957 to 0.995). After adjusting for potential publication bias with the trim-and-fill method, the effect size remains unchanged, reinforcing the robustness of the observed association.

Ahn, J. V., Sera, F., Cummins, S., & Flouri, E. (2018). Associations between objectively measured physical activity and later mental health outcomes in children: findings from the UK Millennium Cohort Study. *J Epidemiol Community Health*, *72*(2), 94-100. <https://doi.org/10.1136/jech-2017-209455>

Bell, S. L., Audrey, S., Gunnell, D., Cooper, A., & Campbell, R. (2019). The relationship between physical activity, mental wellbeing and symptoms of mental health disorder in adolescents: a cohort study. *Int J Behav Nutr Phys Act*, *16*(1), 138. <https://doi.org/10.1186/s12966-019-0901-7>

Booth, J. N., Ness, A. R., Joinson, C., Tomporowski, P. D., Boyle, J. M. E., Leary, S. D., & Reilly, J. J. (2023). Associations between physical activity and mental health and behaviour in early adolescence. *Mental Health and Physical Activity*, *24*, Article 100497. <https://doi.org/10.1016/j.mhpa.2022.100497>

Clare Hume, A. T., Jenny Veitch, Jo Salmon, David Crawford, Kylie Ball. (2011). Physical activity, sedentary behavior, and depressive symptoms among adolescents. *Journal of Physical Activity and Health*, *8*, 152 -156.

da Costa, B. G., Bruner, B., Raymer, G. H., Benson, S. M. S., Chaput, J. P., McGoey, T., Rickwood, G., Robertson-Wilson, J., Saunders, T. J., & Law, B. (2022). Association of daily and time-segmented physical activity and sedentary behaviour with mental health of school children and adolescents from rural Northeastern Ontario, Canada. *Frontiers in psychology*, *13*, Article 1025444. <https://doi.org/10.3389/fpsyg.2022.1025444>

da Costa, B. G., Chaput, J. P., Lopes, M. V. V., Malheiros, L. E. A., & Kelly, S. S. (2022). Movement behaviors and their association with depressive symptoms in Brazilian adolescents: A cross-sectional study. *Journal of Sport and Health Science*, *11*(2), 252-259. <https://doi.org/https://dx.doi.org/10.1016/j.jshs.2020.08.003>

de Faria, F. R., Barbosa, D., Howe, C. A., Canabrava, K. L. R., Sasaki, J. E., & Dos Santos Amorim, P. R. (2022). Time-use movement behaviors are associated with scores of depression/anxiety among adolescents: A compositional data analysis. *PLoS One*, *17*(12), e0279401. <https://doi.org/10.1371/journal.pone.0279401>

Dumuid, D., Olds, T., Lange, K., Edwards, B., Lycett, K., Burgner, D. P., Simm, P., Dwyer, T., Le, H., & Wake, M. (2021). Goldilocks Days: optimising children's time use for health and well-being [Journal Article]. *Journal of epidemiology and community health*. <https://doi.org/10.1136/jech-2021-216686>

Fairclough, S. J., Clifford, L., Brown, D., & Tyler, R. (2023). Characteristics of 24-hour movement behaviours and their associations with mental health in children and adolescents. *Journal of activity, sedentary and sleep behaviors*, *2*(1), 11.

Fairclough, S. J., Tyler, R., Dainty, J. R., Dumuid, D., Richardson, C., Shepstone, L., & Atkin, A. J. (2021). Cross-sectional associations between 24-hour activity behaviours and mental health indicators in children and adolescents: A compositional data analysis. *J Sports Sci*, *39*(14), 1602-1614. <https://doi.org/10.1080/02640414.2021.1890351>

Farren, G. L., Zhang, T., Gu, X., & Thomas, K. T. (2018). Sedentary behavior and physical activity predicting depressive symptoms in adolescents beyond attributes of health-related physical fitness. *J Sport Health Sci*, *7*(4), 489-496. <https://doi.org/10.1016/j.jshs.2017.03.008>

Ghorbani, S., Afshari, M., Eckelt, M., Dana, A., & Bund, A. (2021). Associations between Physical Activity and Mental Health in Iranian Adolescents during the COVID-19 Pandemic: An Accelerometer-Based Study. *Children (Basel)*, *8*(11). <https://doi.org/10.3390/children8111022>

Haapala, E. A., Leppanen, M. H., Kosola, S., Appelqvist-Schmidlechner, K., Kraav, S. L., Jussila, J. J., Tolmunen, T., Lubans, D. R., Eloranta, A. M., Schwab, U., & Lakka, T. A. (2025). Childhood Lifestyle Behaviors and Mental Health Symptoms in Adolescence. *JAMA Netw Open*, *8*(2), e2460012. <https://doi.org/10.1001/jamanetworkopen.2024.60012>

Hagemann, N., Kirtley, O. J., Lafit, G., Wampers, M., Achterhof, R., Hermans, K. S. F. M., Hiekkaranta, A. P., Lecei, A., Vancampfort, D., & Myin-Germeys, I. (2021). Objectively measured physical activity and symptoms of psychopathology in general population adolescents from the SIGMA cohort. *Mental Health and Physical Activity*, *21*. <https://doi.org/10.1016/j.mhpa.2021.100416>

Hamer, M., Patalay, P., Bell, S., & Batty, G. D. (2020). Change in device-measured physical activity assessed in childhood and adolescence in relation to depressive symptoms: a general population-based cohort study. *J Epidemiol Community Health*, *74*(4), 330-335. <https://doi.org/10.1136/jech-2019-213399>

Hrafnkelsdottir, S. M., Brychta, R. J., Rognvaldsdottir, V., Gestsdottir, S., Chen, K. Y., Johannsson, E., Guethmundsdottir, S. L., & Arngrimsson, S. A. (2018). Less screen time and more frequent vigorous physical activity is associated with lower risk of reporting negative mental health symptoms among Icelandic adolescents. *PLoS One*, *13*(4), e0196286. <https://doi.org/10.1371/journal.pone.0196286>

Hume., C., Timperio., A., Veitch., J., Salmon., J., Crawford., D., & Ball, K. (2011). Physical Activity, Sedentary Behavior, and Depressive Symptoms Among Adolescents. *Journal of Physical Activity and Health*(8), 152-156.

Johnson, C. C., Murray, D. M., Elder, J. P., Jobe, J. B., Dunn, A. L., Kubik, M., Voorhees, C., & Schachter, K. (2008). Depressive symptoms and physical activity in adolescent girls. *Med Sci Sports Exerc*, *40*(5), 818-826. <https://doi.org/10.1249/MSS.0b013e3181632d49>

Kandola, A., Lewis, G., Osborn, D. P. J., Stubbs, B., & Hayes, J. F. (2020). Device-measured sedentary behaviour and anxiety symptoms during adolescence: a 6-year prospective cohort study. *Psychol Med*, 1-10. <https://doi.org/10.1017/S0033291720004948>

Kandola., Lewis, G., Osborn, D. P. J., Stubbs, B., & Hayes, J. F. (2020). Depressive symptoms and objectively measured physical activity and sedentary behaviour throughout adolescence: a prospective cohort study. *LANCET PSYCHIATRY*, *7*(3), 262-271. <https://doi.org/10.1016/S2215-0366(20)30034-1>

Kjellenberg, K., Ekblom, O., Ahlen, J., Helgadottir, B., & Nyberg, G. (2022). Cross-sectional associations between physical activity pattern, sports participation, screen time and mental health in Swedish adolescents. *BMJ Open*, *12*(8), e061929. <https://doi.org/10.1136/bmjopen-2022-061929>

Kracht, C. L., Pochana, S. S., & Staiano, A. E. (2023). Associations Between Moderate to Vigorous Physical Activity, Sedentary Behavior, and Depressive Symptomatology in Adolescents: A Prospective Observational Cohort Study. *J Phys Act Health*, *20*(3), 250-257. <https://doi.org/10.1123/jpah.2022-0345>

Martikainen, S., Pesonen, A. K., Lahti, J., Heinonen, K., Tammelin, T., Kajantie, E., Eriksson, J., Strandberg, T., & Raikkonen, K. (2012). Physical activity and psychiatric problems in children. *J Pediatr*, *161*(1), 160-162 e161. <https://doi.org/10.1016/j.jpeds.2012.03.037>

Monteagudo, P., Beltran-Valls, M. R., Adelantado-Renau, M., & Moliner-Urdiales, D. (2023). Observational longitudinal association between waking movement behaviours and psychological distress among adolescents using isotemporal analysis: DADOS study. *Journal of sports sciences*, *41*(13), 1290-1298 %@ 0264-0414.

Nyberg, G., Helgadottir, B., Kjellenberg, K., & Ekblom, O. (2023). COVID-19 and unfavorable changes in mental health unrelated to changes in physical activity, sedentary time, and health behaviors among Swedish adolescents: A longitudinal study. *Front Public Health*, *11*, 1115789. <https://doi.org/10.3389/fpubh.2023.1115789>

Parfitt, G., Pavey, T., & Rowlands, A. V. (2009). Children's physical activity and psychological health: The relevance of intensity. *Acta Paediatrica, International Journal of Paediatrics*, *98*(6), 1037-1043. <https://doi.org/http://dx.doi.org/10.1111/j.1651-2227.2009.01255.x>

Slykerman, R. F., Thompson, J. M. D., Coomarasamy, C., Wall, C. R., Waldie, K. E., Murphy, R., & Mitchell, E. A. (2020). Early adolescent physical activity, sleep and symptoms of depression at 16 years of age. *ACTA PAEDIATRICA*, *109*(7), 1394-1399. <https://doi.org/10.1111/apa.15140>

Van Dijk, M. L., Savelberg, H., Verboon, P., Kirschner, P. A., & De Groot, R. H. M. (2016). Decline in physical activity during adolescence is not associated with changes in mental health. *BMC PUBLIC HEALTH*, *16*, 300. <https://doi.org/10.1186/s12889-016-2983-3>

Wiles, N. J., Haase, A. M., Lawlor, D. A., Ness, A., & Lewis, G. (2012). Physical activity and depression in adolescents: cross-sectional findings from the ALSPAC cohort. *Soc Psychiatry Psychiatr Epidemiol*, *47*(7), 1023-1033. <https://doi.org/10.1007/s00127-011-0422-4>

Yang, L., Corpeleijn, & Hartman. (2023). A prospective analysis of physical activity and mental health in children: the GECKO Drenthe cohort. *Int J Behav Nutr Phys Act.*, *1*(20). <https://doi.org/doi>: 10.1186/s12966-023-01506-1.

Zahl, T., Steinsbekk, S., & Wichstrom, L. (2017). Physical activity, sedentary behavior, and symptoms of major depression in middle childhood. *Pediatrics*, *139*(2), e20161711. <https://doi.org/http://dx.doi.org/10.1542/peds.2016-1711>
